# Heparin Differentially Impacts Gene Expression of Stromal Cells from Various Tissues

**DOI:** 10.1038/s41598-019-43700-x

**Published:** 2019-05-10

**Authors:** Sandra Laner-Plamberger, Michaela Oeller, Rodolphe Poupardin, Linda Krisch, Sarah Hochmann, Ravi Kalathur, Karin Pachler, Christina Kreutzer, Gerrit Erdmann, Eva Rohde, Dirk Strunk, Katharina Schallmoser

**Affiliations:** 1 Spinal Cord Injury and Tissue Regeneration Center Salzburg, Paracelsus Medical University of Salzburg, Salzburg, Austria; 2 Department of Transfusion Medicine, Paracelsus Medical University of Salzburg, Salzburg, Austria; 3 Cell Therapy Institute, Paracelsus Medical University of Salzburg, Salzburg, Austria; 4 GMP Unit, Paracelsus Medical University of Salzburg, Salzburg, Austria; 5 Institute for Experimental Neuroregeneration, Paracelsus Medical University of Salzburg, Salzburg, Austria; 6 Department for Biomedicine, University of Basel, Basel, Switzerland; 7 NMI TT Pharmaservices, Berlin, Germany

**Keywords:** Gene expression, Mesenchymal stem cells

## Abstract

Pooled human platelet lysate (pHPL) is increasingly used as replacement of animal serum for manufacturing of stromal cell therapeutics. Porcine heparin is commonly applied to avoid clotting of pHPL-supplemented medium but the influence of heparin on cell behavior is still unclear. Aim of this study was to investigate cellular uptake of heparin by fluoresceinamine-labeling and its impact on expression of genes, proteins and function of human stromal cells derived from bone marrow (BM), umbilical cord (UC) and white adipose tissue (WAT). Cells were isolated and propagated using various pHPL-supplemented media with or without heparin. Flow cytometry and immunocytochemistry showed differential cellular internalization and lysosomal accumulation of heparin. Transcriptome profiling revealed regulation of distinct gene sets by heparin including signaling cascades involved in proliferation, cell adhesion, apoptosis, inflammation and angiogenesis, depending on stromal cell origin. The influence of heparin on the WNT, PDGF, NOTCH and TGFbeta signaling pathways was further analyzed by a bead-based western blot revealing most alterations in BM-derived stromal cells. Despite these observations heparin had no substantial effect on long-term proliferation and *in vitro* tri-lineage differentiation of stromal cells, indicating compatibility for clinically applied cell products.

## Introduction

The therapeutic potential of various types of human ‘mesenchymal’ stromal cells is currently tested in more than 800 studies (registered at www.clinicaltrials.gov), mainly targeting bone and cartilage regeneration, autoimmune diseases and cardiovascular and neurological disorders^1^. Based on the current debate in the scientific community about the mesodermal origin and tissue specificity of the various stromal cells^2,3^ the term ‘mesenchymal’ referring to an embryonic tissue will be abandoned in this report. Acknowledging the fact that most cells within ‘MSC’ preparations from different tissue are not ‘bona fide’ stem cells, we follow recent recommendations^2,3^ to more correctly term our cells ‘stromal cells’ (STCs), in addition to specifying their organ of origin. Stromal cells from various tissue origins are differing in their transcriptional profile^4^ as well as differentiation potential and DNA methylation signature^5^, influencing biological properties^6^.

For clinical application, a prior *ex vivo* expansion of stromal cells is usually necessary. In the majority of clinical studies fetal bovine serum (FBS) is used as medium supplement^7,8^, despite the risks of transmission of bovine pathogens and xeno-immunization. The European Medicine Agency (EMA) has discouraged the use of animal-derived components for manufacturing of cell-based medicinal products^9^. Alternatively, pooled human platelet lysate (pHPL) is now increasingly used for efficient expansion of stromal cells (for review)^10^. Due to abundant growth factors and cytokines released from various platelet granules^8,11^, pHPL has been confirmed as a suitable replacement for FBS during stromal cell isolation and culture from different tissues, e.g. bone marrow (BM), umbilical cord (UC) or white adipose tissue (WAT)^12–15^.

To avoid clotting of pHPL-supplemented cell culture medium, induced by plasma-derived fibrinogen, prior addition of 0.6–2 IU of porcine heparin per mL medium is common practice^8^. This necessity is hampering completely xeno-free cell culture conditions. However, porcine heparin has been used clinically for several decades by now as anticoagulant to prevent and treat thrombosis and pulmonary embolism^16–18^, as bioengineered human alternatives for this highly sulfated glycosaminoglycan^19,20^ are not yet available for practical use^21^. Nonheparin synthetic anticoagulants inhibiting thrombin, such as fondaparinux, argatroban, or the recombinant hirudin derivatives lepirudin and desirudin are clinically used for the treatment of heparin-induced thrombocytopenia^22^, but their use with HPL for cell culture has not been studied so far.

Proteoglycans are supposed to influence biological processes by interacting with fibroblast growth factors (FGFs), vascular endothelial growth factor (VEGF), or transforming growth factor-beta (TGFbeta)^23,24^. In BM-derived stromal cells (BM-STCs) heparan sulfate significantly upregulated genes involved in cell adhesion and proliferation^25^. Ling *et al*. showed that heparin affected multiple cell signaling components of human BM-STCs in a donor-dependent manner^26^. In these studies, human cells were cultured under FBS-supplemented conditions.

A putative dose-dependent negative effect of heparin was shown on proliferation of vascular smooth muscle cells^27,28^ and stromal cells^26,29^. However, several studies demonstrated that heparin in hydrogels and other biomaterials retained combinations of growth factors and extracellular matrix proteins, supporting proliferation, cell adhesion and immunomodulatory properties of stromal cells^30–32^. Therefore, a benefit of heparin for stromal cell propagation and clinical application is still under debate.

Aim of this study was to investigate the intracellular uptake of heparin into stromal cells derived from BM, UC and WAT, and to analyze the tissue-specific influence of heparin on the gene expression and protein profiles and on biological properties such as proliferation, clonogenicity and *in vitro* differentiation. To enable heparin-free cell culture, fibrinogen was depleted mechanically from pHPL-based medium as described^33^ and stromal cells were cultured in the presence and absence of heparin. By flow cytometry and immunocytochemistry a distinct cellular internalization of fluoresceinamine-labeled heparin mainly in the lysosomal compartment could be detected as described previously for other cell types^34–38^. Comparing gene and protein expression profiles of stromal cells from BM, UC and WAT in the presence and absence of heparin we observed distinct significantly influenced sets of genes, signaling cascades and proteins as well as posttranslational phosphorylation of proteins associated with WNT, PDGF, NOTCH and TGFbeta signaling pathways. Although heparin affected mainly pathways related to proliferation, cell adhesion and regulation of the cytoskeleton, angiogenesis and inflammatory responses, the isolation and long-term propagation as well as *in vitro* tri-lineage differentiation of stromal cells was unaffected by heparin.

## Results

### The canonical fibroblastoid stromal cell immunophenotype is independent of tissue source and heparin

For primary isolation and culture of BM-, UC- and WAT-derived stromal cells, three different pHPL-based media were used: (1) standard pHPL-medium containing fibrinogen and heparin (+fib/+hep), (2) fibrinogen-depleted pHPL-medium without heparin (−fib/−hep) or (3) fibrinogen-depleted pHPL-medium with heparin (−fib/+hep). Independent of cellular exposition to fibrinogen or heparin, flow cytometry analysis revealed the characteristic pattern^39^ CD73^+^/90^+^/105^+^ and CD14^−^/19^−^/34^−^/45^−^/HLA-DR^−^ for all cell types (Supplementary Fig. S1).

### Stromal cells internalize heparin in a source-dependent manner

**Figure 1:**
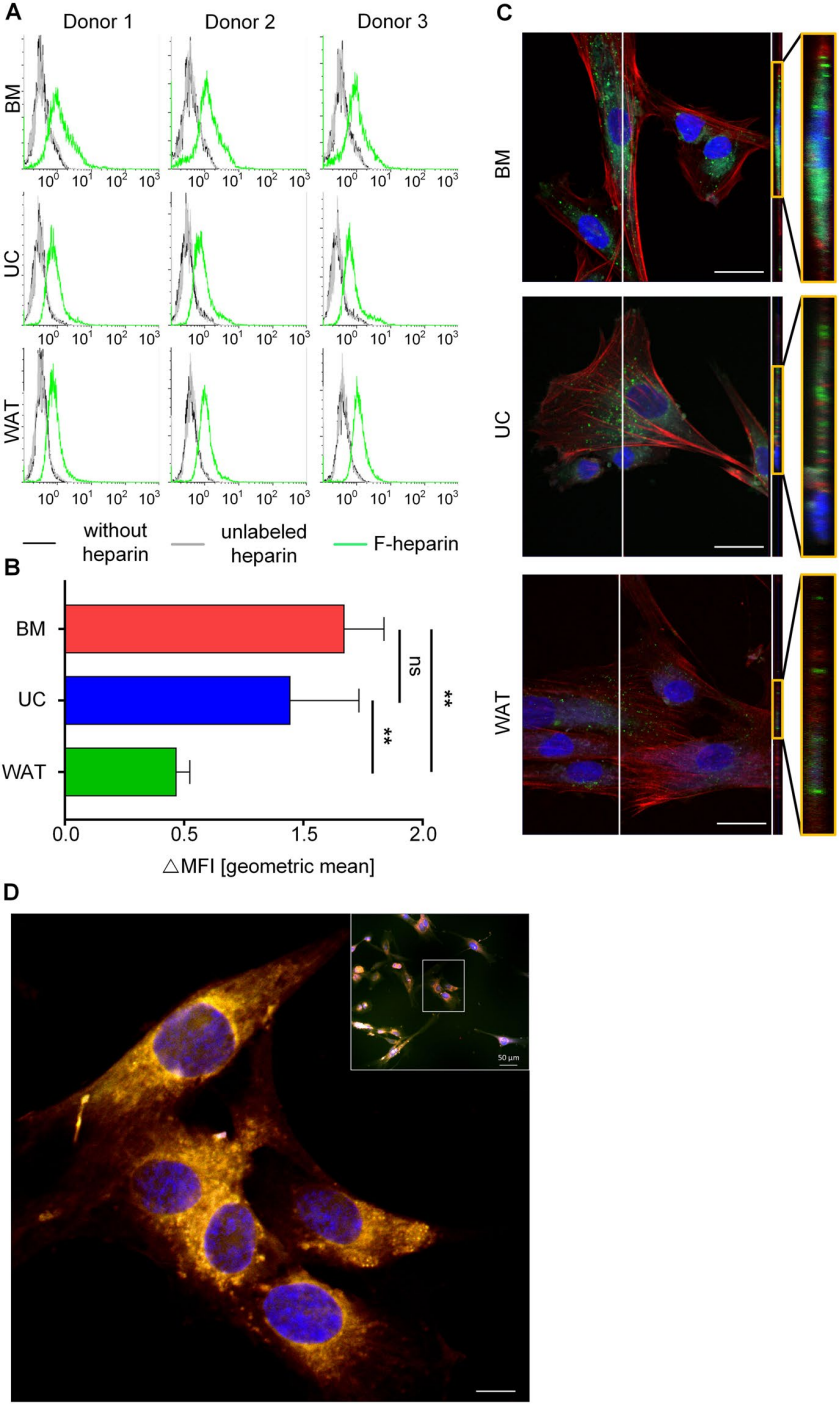
Cellular uptake of heparin in a stromal cell source-dependent manner. (**A**) Flow cytometry analysis of stromal cells cultured without heparin (black), with unlabeled heparin (grey) or fluoresceinamine-labeled heparin (F-heparin, green). (**B**) The results of flow cytometry analysis are depicted as the difference in geometric mean fluorescence intensity (ΔMFI) compared to unlabeled heparin fluorescence, summarized as mean of three independent donations for each tissue source (n = 3). ΔMFI value was significantly lower for WAT- compared to BM and UC stromal cells (*p < 0.05, **p < 0.01). (**C**) Z-stack images of immunofluorescence and orthogonal projections of the z-stacks of representative BM-, UC- and WAT-STCs. Data from one representative donor for each cell source are shown. Scale bars = 10 µm. (**D**) Overlay of z-stack images of UC-derived STCs treated with F-Heparin (labelled green) and LysoTracker (labelled red) simultaneously. The lysosomes appear in an orange color indicating co-localization of F-heparin with lysosomes. Scale bar = 10 µm.

As heparin uptake has been observed for other cell types such as endothelial cells^35^, lymphocytes^36^, monocytes^37^ and different cancer cells^34^, we asked whether heparin is differentially internalized by stromal cells from various tissues. The different stromal cell types were incubated with fluoresceinamine-labeled heparin (F-heparin) as described previously for endothelial cells and various cancer cell lines^34^. The uptake of F-heparin was compared to culture conditions without heparin or with unlabeled heparin. Flow cytometry showed that stromal cells differentially internalized heparin (Fig. 1A) depending on cell origin. BM- and UC-STCs internalized significantly more heparin molecules than WAT-STCs (Fig. 1B). Z-stack images of cells cultured with F-heparin were done using a confocal laser microscope. Orthogonal projection of the confocal images confirmed cellular uptake of F-heparin (Fig. 1C), whereas no F-heparin was detected on the cell surface. In BM- and UC-STCs F-heparin aggregates were clearly localized close to the nuclei. In accordance to the results of flow cytometry, substantially less F-heparin internalization was observed for WAT-STCs (Fig. 1C). Since data exist indicating that heparin is internalized by lysosomes^38,40^, we next incubated UC-derived stromal cells simultaneously with F-heparin and LysoTracker. Z-stack images obtained by confocal laser microscopy indicated that heparin’s intracellular distribution was associated with lysosomes (Fig. 1D and Supplementary Fig. S2A,B).

### Heparin influences gene expression differentially depending on the cell source

**Figure 2:**
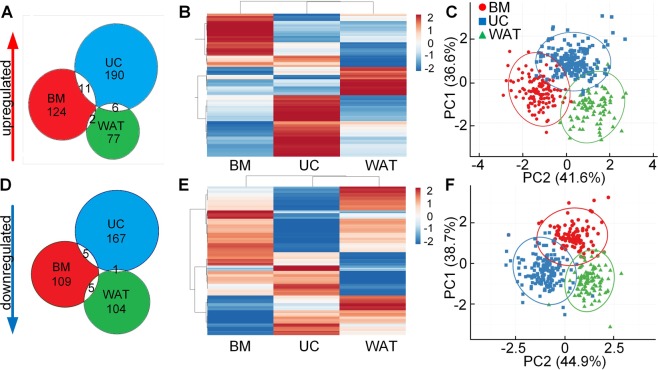
Regulation of distinct gene sets by heparin. Gene expression profiling of WAT, UC or BM stromal cells cultured in the absence of fibrinogen and heparin (−fib/−hep) compared to culture with heparin (−fib/+hep). (**A**,**D**) Venn diagrams depict the number of genes that were (**A**) significantly upregulated or (**D**) significantly downregulated by heparin with a fold change of at least +/− 1.5 in all three independent biological samples for each tissue source (p < 0.05). (**B**,**E**) Heatmaps illustrate expression analysis data of substantially (**B**) upregulated or (**E**) downregulated genes. The top 15 upregulated genes for each tissue source are listed in Supplementary Table S1, the top 15 downregulated are shown in Supplementary Table S2. (**C**,**F**) Principal component analysis (PCA) for (**C**) upregulated and (**F**) downregulated genes applying singular value decomposition with imputation. Prediction ellipses show that with a probability of >95% the gene expression profile of heparin-exposed stromal cells of an additional donor will fall inside the corresponding ellipse.

Heparan sulfates and heparin were described as important co-factors for several cell signaling pathways^41^, but the influence with respect to gene expression and different cell sources is poorly understood. We therefore used three individual donations from BM, UC and WAT for whole genome expression profiling and compared stromal cells cultured in the absence of fibrinogen and heparin (−fib/−hep) compared to culture with heparin (−fib/+hep). Genes were considered as significantly regulated by heparin if gene expression revealed a fold change of ±1.5 and a p-value ≤0.05 for each of the three donations per tissue. Our data revealed that heparin affected the expression of 410 genes substantially in a tissue dependent manner (Fig. 2A). UC-STCs showed the highest number of genes upregulated by heparin with 207 genes compared to 137 genes in BM-, and 85 genes in WAT-STCs. Interestingly, no common genes were significantly upregulated by heparin in all the three sources of cells. Only a few genes were significantly upregulated by heparin in two of the three tissue sources, i.e. showed an overlap between two of the tissue sources:

- *SLC25A3* and *ANGPT1* in BM- and WAT-STCs;
- *MIR326*, *ITGB8*, *CFB*, *BMP4*, *CFI*, *SEPP1*, *SVEP1*, *ANKRD29*, *GUCY1B3*, *YY2* and *TPTE2P6* in BM- and UC-STCs and
- *CLEC18A*, *ARHGEF3*, *SPN*, *MIR944*, *YME1L1* and *LOC101928303* in UC- and WAT-STCs.
  These findings are also reflected by a heatmap (Fig. 2B): All genes significantly upregulated by heparin in UC-STCs showed a significantly different expression pattern in WAT- or BM-STCs. The same was observed for genes substantially upregulated in BM- and WAT-STCs.
  A similar effect of heparin was found for gene expression downregulation. The highest number of genes (173) significantly downregulated by heparin was found for UC-STCs, 119 genes were downregulated in BM- and 110 genes in WAT-STCs. No genes were significantly downregulated in all three cell sources (Fig. 2D). Only a small set of genes was significantly downregulated in two of the three sources:
- *MIR3619* in UC- and WAT-STCs,
- *PANX2*, *DSP*, *ID3*, *MYOCD* and *CALB2* in UC- and BM-STCs,
- *LOC101927993*, *NOTCH3*, *SNORA46*, *MFAP3* and *LINC00862* in WAT- and BM-STCs.

In Fig. 2E the heatmap reflects the strictly source-dependent effect of heparin on downregulation of gene expression. A principal component analysis including all genes (Fig. 2C) upregulated and (Fig. 2F) downregulated by heparin confirmed the statistical significance of our data. Prediction ellipses indicate that the gene expression profile of heparin-exposed stromal cells of an additional donor will fall inside the corresponding ellipse with a probability of >95%.

### Heparin regulates distinct signaling pathways

**Figure 3:**
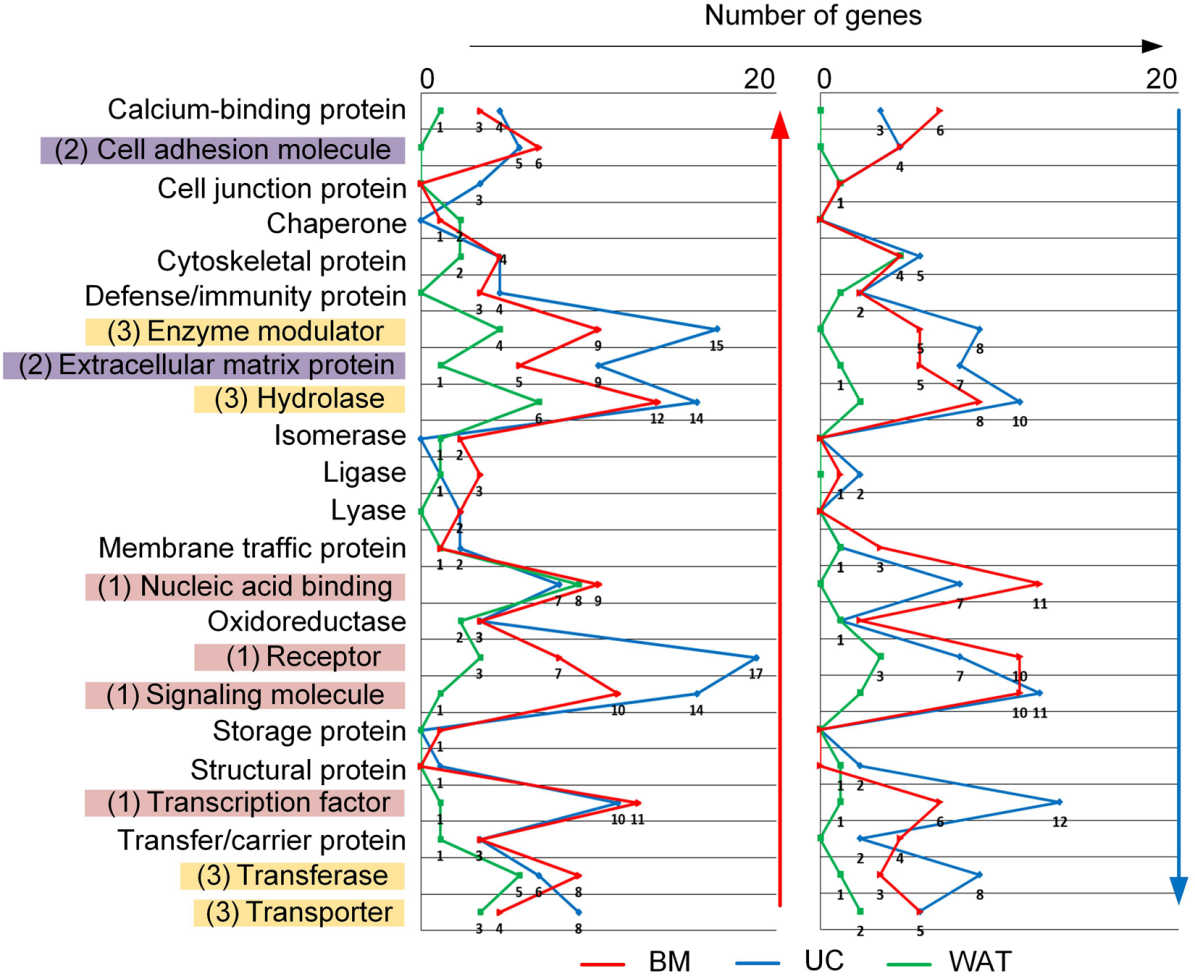
Different molecule classes affected by heparin. Classification of heparin-regulated genes coding for protein classes showed that most molecules were either (1) proteins associated with cell signaling including proteins with nucleic acid binding properties, receptors, signaling molecules and transcription factors (marked in red), (2) extracellular matrix proteins and proteins associated with cell adhesion (marked in violet) or (3) enzyme modulators, hydrolases and transferases, but also proteins with transporter functions (marked in yellow). The indicated color code depicts cell sources, respectively. Number of genes per molecule class and data point have been inserted. Red and blue arrows indicate up- and downregulation, respectively.

To gain insight into the biological activity of the genes significantly regulated by heparin, a clustering according to molecule classes was performed (Fig. 3). Genes regulated by heparin were found to code mainly for proteins of cell signaling pathways (such as receptors, signaling molecules, transcription factors and proteins with nucleic acid binding properties), cell adhesion molecules and proteins of the extracellular matrix as well as enzyme modulators and proteins with hydrolase activity or proteins being involved in cell metabolism. Supplementary Tables S1 and S2 show the top 15 protein-coding genes being either up- or downregulated by heparin sorted according to their fold change value.

**Figure 4:**
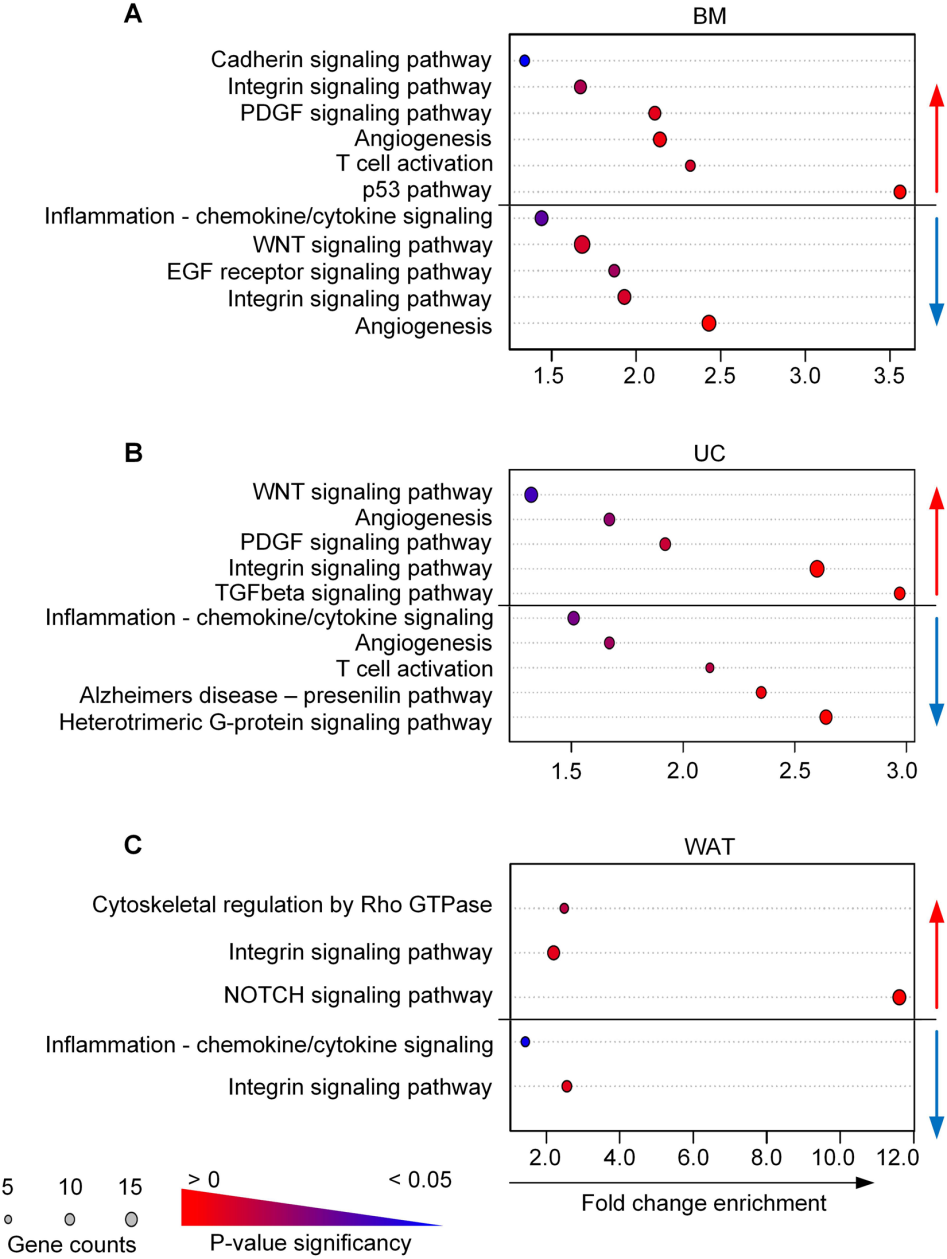
Heparin modulated signaling cascades and pathways. Significantly regulated genes were assigned to signaling pathways using R and Panther analysis. Dot blots show significantly regulated pathways for (**A**) BM, (**B**) UC and (**C**) WAT stromal cells. Pathways with statistically considerable overrepresentation after heparin treatment are depicted by gradient color code of the dots whereby highest significance p-value is marked as red (p > 0) and decreases to blue (p < 0.05). The size of each dot correlates with the number of genes assigned to each pathway of the term list as indicated. Red and blue arrows indicate up- and downregulation, respectively. Dependent on the cell source different sets of signaling pathways were affected by heparin with the lowest influence on WAT stromal cells.

Functional enrichment analysis revealed that each stromal cell source showed distinct pathways regulated by heparin (Fig. 4). BM- and UC-STCs showed a significant regulation of members of the WNT and PDGF pathway (e.g. *FZD2*, *FZD5*, *FZD10*, *WNT2*, *SMAD1*, *PDGFD*, *JAK3*). While TGFbeta signaling was upregulated by heparin-exposed UC-STCs, p53- and T-cell activation were significantly enhanced in BM-STCs (Fig. 4A,B). Members of the NOTCH signaling pathway (*RBPJL*, *APH1A*, *HEYL*, *HELT*, *MFNG*, *NEURL1*, *HES5*) were upregulated in WAT-STCs, whereas none of these genes was significantly regulated in BM- or UC-STCs (Fig. 4A–C). Furthermore, genes and pathways associated with cell adhesion and regulation of the cytoskeleton such as integrin, cadherin signaling and cytoskeletal regulation by Rho-GTPase were upregulated by heparin in all cell types.

Heparin also negatively affected the expression of distinct pathways. Inflammatory pathways and cytoskeletal genes mediating cell-cell interactions during inflammatory responses or activation of lymphocytes such as *ITGAX* and *ITGAE* as well as negative regulators of WNT, NOTCH and EGFR pathway such as *GREM1/2*, *APC2*, *NLK* and *CBL* were significantly downregulated by heparin.

**Figure 5:**
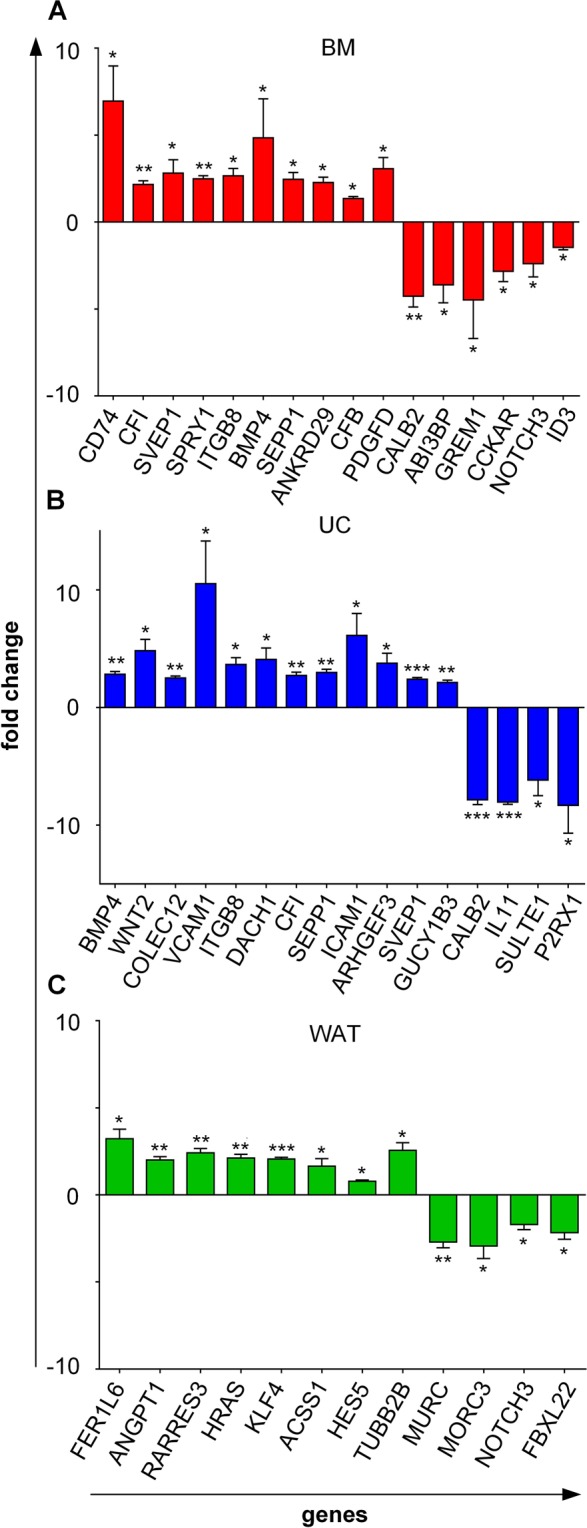
Confirmation of heparin target gene expression. Quantitative RT-PCR of selected target genes conducted for differentially regulated genes in response to heparin in (**A**) BM, (**B**) UC and (**C**) WAT stromal cells. Data shown are mean fold-changed values of three individual donors for each tissue source measured in duplicates, comparing gene expression in fibrinogen-depleted culture conditions with or without heparin (−fib/−hep versus −fib/+hep). Unpaired t-test was used to determine statistically significant differences (*p < 0.05, **p < 0.01, ***p < 0.001).

Quantitative RT-PCR was conducted for selected target genes of each cell source and confirmed the findings of genome expression analysis (Fig. 5A–C and Supplementary Table S3).

### Heparin differentially affects the WNT, PDGF, NOTCH and TGFbeta signaling pathways at the protein level

**Figure 6:**
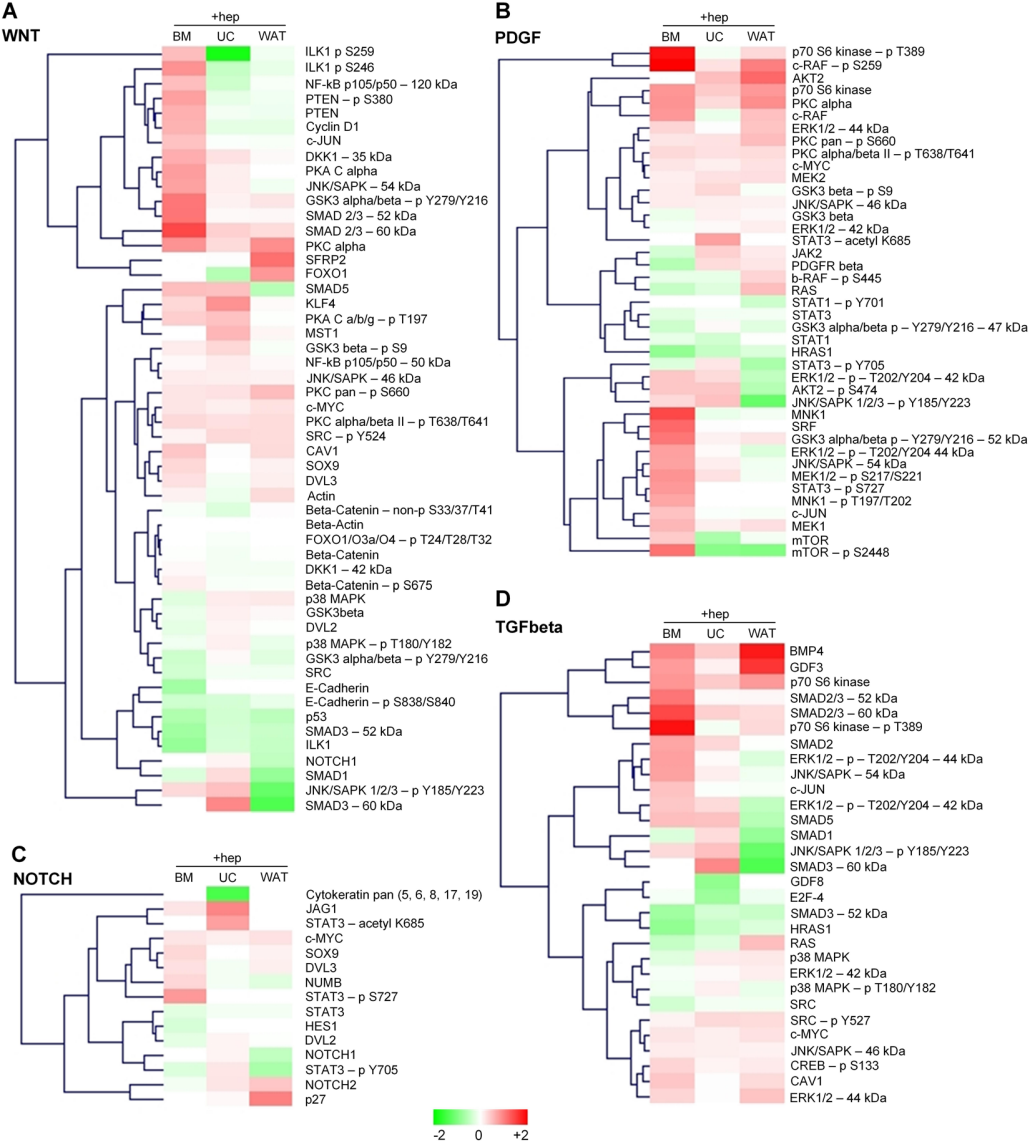
Heparin differentially affects protein expression levels of WNT-, PDGF-, NOTCH- and TGFbeta signaling pathways. The heatmaps of (**A**) WNT-, (**B**) PDGF-, (**C**) NOTCH- and (**D**) TGFbeta-pathways reflect the positive and negative ratios of protein expression of BM-, UC- and WAT-derived stromal cells with and without heparin exposure (depicted as +hep) with red color indicating over- and green color under-representation compared to cells without heparin. Cells were cultured as described in the legend to Fig. 5, subjected to DigiWest multiplexed protein profiling and subsequent data analysis as described in the methods section.

A bead-based western blot (DigiWest)^42^ was performed to analyze the influence of heparin on the protein expression and posttranslational phosphorylation of proteins associated with WNT, PDGF, NOTCH and TGFbeta signaling pathways. A total of 103 different antibodies were used to analyze the expression levels for one donor per tissue source. In sum, 97 targets of the analyzed proteins of the four pathways were differentially expressed in BM-, UC- and WAT-derived stromal cells, as shown by MFI values and expression patterns compared to the housekeeping protein beta-actin (Supplementary Fig. S3). To compare protein expression levels in the absence or presence of heparin, the ratio was calculated and depicted as heat maps of the four analyzed pathways (Fig. 6A–D). A difference of more than 20% was defined as substantial effect of heparin on protein expression. In general, more proteins were upregulated in response to heparin than downregulated and compared to the other sources BM-STCs showed the highest number of altered proteins (Fig. 6A–D and Supplementary Tables S4 and S5).

Confirming gene expression analysis, a tissue-specific expression was observed for most of the protein targets (Supplementary Fig. 3), e.g., expression of the TGFbeta pathway member protein SMAD1 was upregulated in UC-derived stromal cells, but not in BM- or WAT-STCs. In contrast, the BMP4 protein was upregulated in all sources. The WNT signaling member PTEN showed elevated protein expression and phosphorylation status in BM-, but not in UC- or WAT-derived stromal cells. Further WNT signaling members with differentially enhanced protein expression in response to heparin were CyclinD1, JNK/SAPK, KLF4, NK-kB, PKC and SMAD5. Even though in WAT-STCs the NOTCH signaling gene expression was influenced by heparin, this could not be corroborated on the protein level, except for NOTCH2. Interestingly, the expression of ERK1/2 was unchanged in BM-STCs in response to heparin, whereas the phosphorylation status of certain amino acids was enhanced. A similar observation was made for PDGF pathway member AKT2 and WNT members ILK1 and GSK3-beta (for more details see Supplementary Fig. 3).

### Only the short-term proliferation rate of stromal is sustainably influenced by heparin

Our data revealed that WNT, PDGF and NOTCH signaling pathways, known to affect stromal cell proliferation^43–46^, were significantly regulated by heparin. We therefore investigated the proliferative capacity of stromal cells in the presence and absence of heparin by calculation of Δ proliferation compared to standard pHPL culture with fibrinogen and heparin and by comparing cumulative population doublings (Supplementary Fig. S4). In passage one, the proliferation of BM-STC was significantly reduced without fibrinogen and enhanced by addition of heparin (Supplementary Fig. S4A). As observed previously^33^, heparin also significantly enhanced proliferation of UC-STCs in the absence of fibrinogen in passage one and two (Supplementary Fig. S4C). The proliferation of WAT-STCs was independent of fibrinogen and heparin (Supplementary Fig. S4E). However, there was no significant sustainable effect of heparin on the cumulative population doublings of the three cell sources tested over three passages (Supplementary Fig. S4B,D,F).

As the abundant amount of growth factors in pHPL may cover an effect of heparin on cell proliferation, we reduced the pHPL concentration by titration (10%, 3.3% and 1.1%) in the cell culture medium and tested the impact of 2 and 4 IU/mL heparin on cell proliferation of stromal cells from UC, BM and WAT. As shown in the Supplementary Fig. S5 the mean total cell numbers of stromal cells from the three tissues did not differ significantly in the various cell culture conditions within the observation period.

To exclude specific effects of F-heparin on proliferation for the immunofluorescence experiment, we also compared the effect of unlabeled heparin and F-heparin by XCELLIgence impedance analysis. No significant differences were observed (Supplementary Fig. S6).

### Colony forming capacity of stromal cells is partially influenced by fibrinogen but independent of heparin

In passage one, fibrinogen caused significantly enhanced cloning efficiency of BM-STCs (Supplementary Fig. S7A,B; p < 0.01), in contrast significantly reduced cloning efficiency of UC-STCs (Supplementary Fig. S7C,D; p < 0.01), but had no effect on WAT-STCs (Supplementary Fig. S7E,F). Except for a significant increase of colony numbers of UC-STCs in passage 1 (Supplementary Fig. S7C,D; p < 0.01) the addition of heparin had no effect on cloning efficiency of stromal cells of all tissues.

### *In vitro* differentiation of stromal cells is independent of heparin

**Figure 7:**
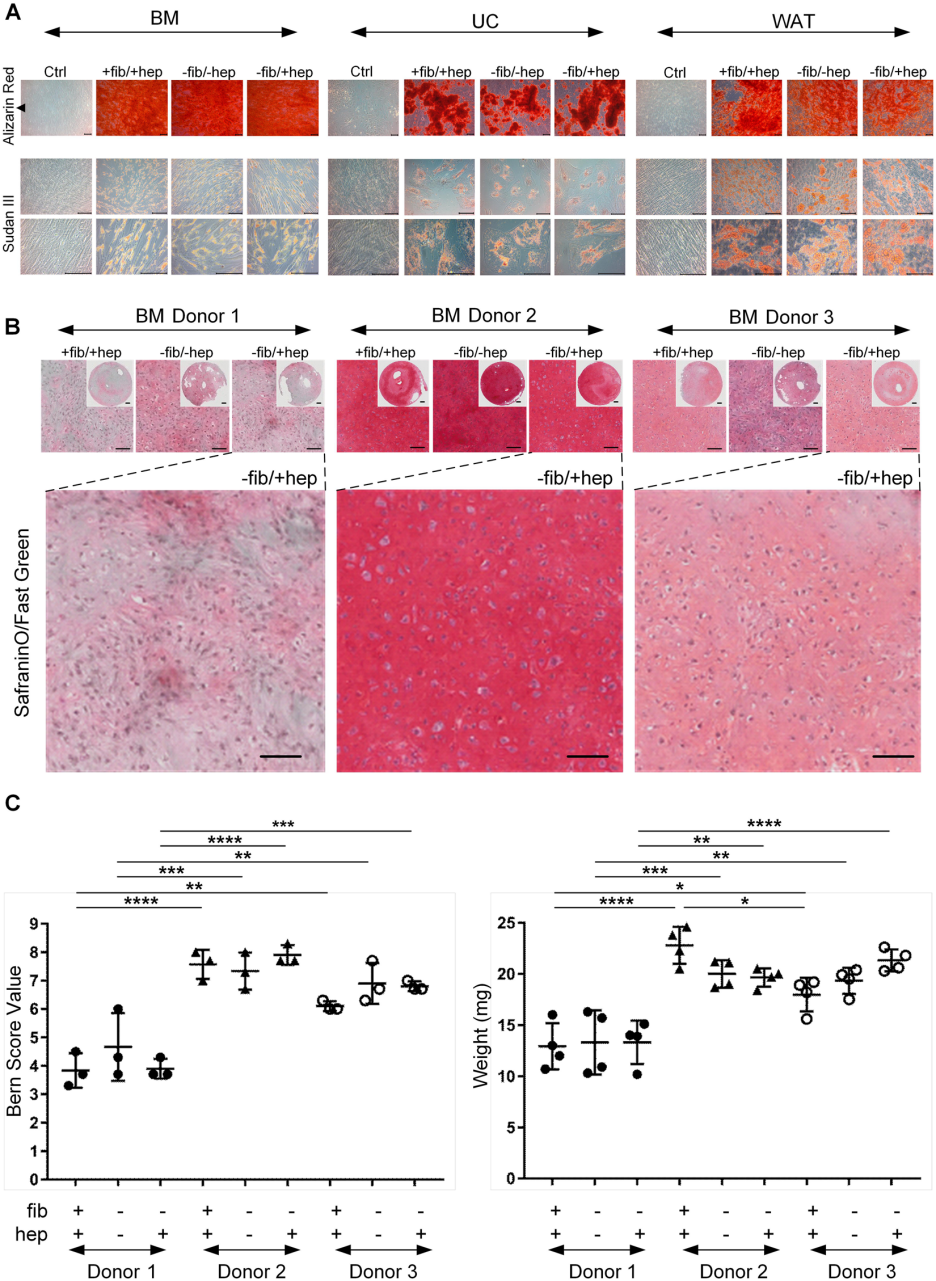
*In vitro* differentiation of stromal cells. (**A**) *In vitro* osteogenic and adipogenic differentiation of one representative donor out of three tested for each tissue source in different culture media are displayed as indicated. Magnification: 40x for osteogenic differentiation and corresponding control (Alizarin Red staining) and 100x and 200x for adipogenic differentiation and control (Sudan III staining). (**B**) *In vitro* 3D chondrogenic differentiation was conducted for BM stromal cells. SafraninO/Fast Green staining indicate *in vitro* chondrogenesis. Scale bar: 100 µm. (**C**) Results were evaluated by Bern Scoring^47^ and by measuring the weight of the 3D cartilage discs (*p < 0.05, **p < 0.01, ***p < 0.001, ****p < 0.0001).

The *in vitro* osteogenic and adipogenic differentiation potential was independent of cell source or culture conditions (Fig. 7A). Since WAT- and UC-STCs were previously shown not to differentiate into chondrogenic lineages *in vivo*^2,4,5^, chondrogenic differentiation was analyzed for BM-STCs (n = 3) only. Cartilage discs were generated by 3D transwell cultures and stained with SafraninO/Fast Green (Fig. 7B). For independent evaluation a visual histological grading system was applied (Bern Score)^47^ and the weights of the 3D cartilage discs were measured in addition (Fig. 7C). SafraninO/Fast Green staining, Bern Scoring and cartilage discs weights revealed no significant differences between the culture conditions, whereas substantial donor-dependent differences were observed. In summary, *in vitro* tri-lineage differentiation of stromal cells was found unaffected by fibrinogen and heparin.

## Discussion

Human stromal cells are key candidates for cell therapy to treat a wide variety of diseases^1^. Although international regulatory authorities discourage the use of animal-derived components for manufacturing of stromal cell-based medicinal products, still most protocols for cell isolation and propagation use bovine serum or other animal components^7^. The use of pHPL-supplemented media has been proven for stromal cell expansion whereas the addition of porcine heparin is common practice for anticoagulation of the media. Currently, no human alternatives for porcine heparin are commercially available^21^. We therefore studied the putative impact of porcine heparin on gene and protein expression as well as biological properties of stromal cells from various tissues.

After the primary isolation of stromal cells in different pHPL-based media, flow cytometry revealed the characteristic immunophenotype for cells of all tissue sources. An internalization of heparin and heparin-like polysaccharides has been shown for tumorigenic and non-tumorigenic cell types^34^. The authors reported that the sulfation pattern of the glycosaminoglycan molecules and the cell type determined the cellular localization of heparin and heparin-like molecules. We therefore analyzed the cellular uptake of labeled heparin for all three stromal cell types tested in this study. Flow cytometry analysis revealed that the cellular uptake of fluorescently labelled heparin was dependent on the tissue origin and was significantly higher in BM- and UC-STCs compared to WAT-STCs. These results were confirmed by immunofluorescence showing that the intracellular distribution of heparin was associated to lysosomes. Our observations go ahead with previous data in the literature about the systemic clearance of heparin from the circulation mediated by a hyaluronan receptor for endocytosis (HARE), derived from stabilin-2 by proteolysis^48,49^. This primary scavenger receptor had an affinity not only for hyaluronic acid and chondroitin sulfate but also for heparin and other ligands, binding and internalizing these macromolecules for further degradation in lysosomes^40^. HARE has been shown to be highly expressed on sinusoidal endothelial cells of liver and lymph node, spleen, and bone marrow, but also macrophages and mesenchymal heart valve cells (for review)^48^. Systemic clearance receptors are supposed to be in a dynamic recycling pathway just localizing to coated pits of the plasma membrane. Therefore, less than 30% of the total clearance receptors are detectable on the cell surface hampering the detectability of this receptor on the surface of stromal cells.

In our study, a concentration of 2 IU heparin per mL medium significantly altered gene expression profiles already at early passages and in a strictly source-dependent manner. Comparing the global gene expression patterns of BM-, UC- and WAT-STCs cultured in fibrinogen-free medium with or without heparin, we identified significant changes in gene expression in all cell types already at passage one, whereas previous data from Ling *et al*. showed heparin-induced long-term alterations of gene expression of BM-derived stromal cells cultured in FBS at later passages (4–8)^26^. In our study gene expression of UC-STCs was more sensitive to heparin compared to BM- or WAT-STCs. UC-STCs showed the highest numbers of significantly regulated genes and higher fold changes of gene expression. Gene expression array analysis and confirmative qRT-PCR of selected target genes indicated that heparin significantly regulated distinct sets of genes in a strictly source-dependent manner. We found several members of the NOTCH pathway to be significantly upregulated in WAT- but not in BM- or UC-STCs. Ligands, receptors and transcription factors of the WNT-, PDGF- and TGFbeta pathways were significantly upregulated in BM- and UC-STCs only. These pathways play pivotal roles for proliferation of stromal cells^50^.

To prove gene expression changes induced by heparin at the protein level, we performed a bead-based western blot assay to analyze expression and phosphorylation status of proteins of the WNT, PDGF, NOTCH and TGFbeta pathways. We observed differential alterations depending on the cell source. Whereas more proteins were upregulated than downregulated by heparin, most alterations were found in BM-derived stromal cells. Our results are in line with previous reports in literature showing that an activation of global cell signaling pathways including extracellular signal-regulated kinase (ERK1/2) and nuclear factor-κB (NF-κB) signaling were induced by endocytosis of heparin via HARE^51^, indicating a possible influence of heparin on gene expression. Furthermore, a close association between lysosomal degradation and activation of mammalian target of rapamycin (mTOR) and mitogen-activated protein kinase (MAPK) signaling, central signaling pathways for cell growth and survival, integrating signaling from growth factors and nutrients, has been described (for review)^52^. The lack of an impact of heparin on long-term cell proliferation in our study *in vitro* despite significant gene and protein expression changes is obviously surprising but could not be explained so far.

We also identified multiple genes associated with cell adhesion and regulation of the cytoskeleton being significantly upregulated by heparin. Furthermore, negative regulators of proliferative pathways and genes associated with inflammatory processes were significantly downregulated. These findings are in line with that reported by Ling *et al*.^26^. However, our data indicate that changes in gene expression induced by heparin can already be observed after short-term cultivation and that these changes occurred in a cell source-dependent manner.

Regarding the differential cell source dependent cellular heparin internalization and results of gene expression analysis, we investigated the cell proliferation rate and clonogenicity. All stromal cells were efficiently propagated in fibrinogen-depleted medium independent of the presence of heparin. In line with our gene and protein expression analysis, a significant increase of BM- and UC-STC proliferation in early passages was observed. However, proliferation of WAT-STCs and the cumulative population doublings over three passages (long-term proliferation) were unaffected by heparin for all stromal cells.

Also the cloning efficiency was not influenced by heparin, but a source-dependent effect of fibrinogen-depletion was observed. While BM-STCs showed a substantially reduced colony forming capacity, UC-STCs showed an enhanced clonogenicity in the absence of fibrinogen.

The analysis of *in vitro* tri-lineage differentiation confirmed that the exposition of heparin and fibrinogen had no significant influence on the stromal cells, as observed differences were rather donor specific. For note, *in vitro* differentiation assays for stromal cells should be assessed critically in general due to putative artifacts and unspecific staining^2^.

This study has some limitations: (I) Cellular internalization of F-heparin in lysosomes was tissue-dependent but expression of the corresponding HARE on stromal cell surface has not been shown so far. (II) Baseline gene expression of primary naïve stromal cells in the BM, UC or WAT before *in vitro* expansion would be a more precise control for analysis of gene alterations induced by heparin exposition. However, due to low frequency in primary tissue samples a valid analysis was not possible in this study.

Our data indicate that heparin is internalized and degraded in lysosomes by stromal cells in a tissue-source dependent manner, thereby inducing not only differential gene expression but also protein expression and phosphorylation changes. Putative differential post-translational modifications of involved proteins may be the reason that biological properties as immunophenotype, long-term proliferation, clonogenicity and *in vitro* tri-lineage differentiation of stromal cells maintained unaffected by heparin, indicating that application of porcine heparin does not interfere with efficient manufacturing of stromal cell based medicinal products.

## Methods

### Cell culture medium preparation

Pooled HPL (pHPL) was produced as described previously^15,53^ and was used to supplement alpha modified Minimum Essentials Eagle’s Medium (α-MEM, Sigma Aldrich, St. Louis, MO, USA) (10% v/v) for propagation of stromal cells. Three different medium types were generated: (1) pHPL supplemented α-MEM with heparin (2 IU/ml; Biochrom, Berlin, Germany) to avoid clot formation (+fibrinogen/+heparin), (2) pHPL supplemented α-MEM prepared by mechanical fibrinogen-depletion as described previously^33^ (−fibrinogen/−heparin) and (3) pHPL supplemented, mechanically fibrinogen-depleted α-MEM with heparin (−fibrinogen/+heparin). After addition of 5.5 mM (N2)-L-Alanyl-L-Glutamin (Dipeptiven, Fresenius Kabi, Graz, Austria) all media were sterile filtrated before use.

### Culture conditions, isolation and propagation of stromal cells

The study was performed in accordance with the Helsinki Declaration and all protocols were approved by the ethical committee of federal state of Salzburg. All donors of BM, WAT and UC signed an informed consent concerning the research use of materials. Additionally, BM samples were obtained from AllCells, (Alameda, CA, USA, www.allcells.com/statement-of-ethical-standards/). WAT-STCs (n = 3) were isolated according to Zhu *et al*.^54^. UC- and BM-STCs (both n = 3) were isolated as described previously^39,55^. For cell isolation from BM under heparin-free conditions, BM was aspirated and further isolated without any anticoagulant. The clotted aspirate was cultured in fibrinogen-free medium. Antibiotics (100 mg/mL streptomycin and 62.5 mg/mL penicillin, LifeTechnologies, Carlsbad, CA, USA), were used for initial cell isolation from UC only and removed after 48 hours. All subsequent culture conditions lacked antibiotics and cells were cultured at 37 °C and ambient air conditions. For the determination of proliferation rates, differentiation potential and clonogenicity, all stromal cells were cultured in the three 10% pHPL-based medium preparations as described above. To test whether the growth factor levels in 10% pHPL-based medium may mask an effect of heparin, stromal cells were also cultivated in 3.3% and 1.1% pHPL in combination with either 2 or 4 IU/mL heparin (Biochrom).

### Flow cytometry analysis

Purity, identity and viability of stromal cells were characterized by flow cytometry as previously described^39^. In brief, 5 × 10^5^ cells at passage 1 were resuspended in 50 µL phosphate buffered saline (PBS, Sigma Aldrich). Cells were mixed with 0.5 µL viability dye (Fixable Viability Dye eFluor™ 520, eBioscience, Thermo Fisher Scientifics, Waltham, MA, USA) and antibody mastermix for CD73, CD19, CD14, CD34, CD45, HLA-DR (all Becton Dickinson BD Biosciences, Franklin Lakes, NJ, USA), CD90 (Beckman Coulter, Brea, CA, USA) and CD105 (Life Technologies Corporation, Frederick, MD, USA). For determination of F-heparin internalization, 1 × 10^3^ cells per cm^2^ were seeded. After 48 hours, cells were incubated for three hours with medium supplemented with 10% pHPL either without heparin, with 2 IU/ml unlabeled heparin (Biochrom) or with 2 IU/ml fluoresceinamine-labeled sodium heparin (F-heparin, PG Research, Tokyo, Japan). After washing cells twice with PBS, 2 × 10^5^ cells were resuspended in 100 µL 7AAD viability dye mastermix (BD). All cells were measured immediately (BD LSRFortessa™A) and results were analyzed with Kaluza Analysis Software (Beckman Coulter).

### Immunofluorescence

For immunofluorescence, 1 × 10^3^ cells were seeded on glass coverslips (Marienfeld-Superior, Lauda-Königshofen Germany) and incubated as described for flow cytometry analysis. For staining of lysosomes, stromal cells were incubated simultaneously with F-heparin and 60 nM LysoTracker Red DND-99 (Molecular Probes by Life Technologies Corporation) according to the manufacturer’s instructions. After washing twice with PBS, cells were fixed with 4% PFA, washed with PBS and blocked with 2% bovine serum albumin (BSA, Sigma Aldrich) for one hour. Then cells were incubated with Alexa Fluor 568 Phalloidin (Molecular Probes, Eugene, OR, USA) diluted in 0.2% BSA (1:500) for 1 hour at room temperature in the dark. After two washing steps, an incubation with 4′,6-Diamidin-2-phenylindol (DAPI, diluted 1:1000 in 0.2% BSA; Molecular Probes) for 10 minutes at room temperature in the dark was done. Finally, stained cells on glass coverslips were mounted with 40 μL ProLong Gold Antifade (Molecular Probes) on microscope slides (Marienfeld-Superior). For visualization, a Zeiss 710 Confocal Laserscanning Microscope (Zeiss, Oberkochen, Germany) with ZEN black imaging software was used to produce z-stack images of the probes. Pictures shown are orthogonal projections of the z-stacks.

### RNA isolation and microarray analysis

Total RNA was isolated from stromal cells (passage 1) cultured in different pHPL-media using High Pure RNA isolation kit (Roche Diagnostics, Rotkreuz, Switzerland) according to manufacturer’s instructions. Quality of RNA was analyzed by Agilent 2100 Bioanalyzer (Agilent Technologies, Foset City, CA, USA) using Agilent RNA 6000 Nano Kit (Agilent Technologies) according to manufacturer’s protocol. RNA samples with RNA integrity number (RIN) values >9 were subjected to further analysis. Microarray analysis on an Affymetrix Human Gene 2.1 ST array was done with RNA samples of three different donors for each tissue source. All hybridizations were carried out at the Core Facility for Fluorescent Bioanalytics of the University of Regensburg, Germany. Data were analyzed using R (https://www.r-project.org)/Rstudio (https://www.rstudio.com) with Bioconductor and ClusterProfiler add-on packages. The background signal correction, normalization and summarization were performed by Robust Multiarray Averaging^56^ from the affy package^57^. To identify differences in gene expression between treated and control samples, the linear models for microarray data (limma)^58^ package was used. Genes with an adjusted p-value of ≤0.05 and an absolute fold change of ≥1.5 or ≤−1.5 were considered differentially expressed. Heatmaps and principal component analysis (PCA) were done using ClustVis tool^59^.

### Quantitative real-time PCR (qRT-PCR)

Quantitative real-time PCR reactions were done with the same batches of total RNA as were used for microarray analysis. cDNA synthesis was done as described^60^. qRT-PCR analysis was performed using a LightCycler 480 II and LightCycler 480 SYBR Green I Master reagent (both Roche Diagnostics) according to manufacturer’s instructions. Putative heparin target genes were randomly selected to be corroborated by qRT-PCR. Human Glyceraldehyde 3-phosphate dehydrogenase (GAPDH) was used for normalization of sample material. Data analysis was done as described previously^61^. For qRT-PCR primer sequences see Supplementary Table S6.

### DigiWest multiplexed protein profiling

The DigiWest protocol was performed as described^42^. Samples were lysed directly in 6-well plates using LDS containing lysis buffer. Protein perception was performed using the 2-D Clean-Up kit (GE Healthcare, Freiburg, Germany) according to manufactures protocol. Precipitated Protein was solubilized again in 50–100 µL of LDS containing lysis buffer (Life Technologies, Darmstadt, Germany). Protein concentrations were determined using the 660 nm assay (Thermo Fisher Scientific, Schwerte, Germany) according to manufactures protocol. Briefly, SDS-PAGE and Western blotting onto PVDF membranes (Millipore) was performed using the NuPAGE system (Life Technologies) loading 15 µg total protein per sample. Blots were washed in PBS containing 0.1% Tween-20 (PBST) and proteins were biotinylated on the membrane using NHS-PEG12-Biotin (Thermo Fisher Scientific) in PBST. Membranes were then washed in PBST and dried. Each sample lane was dissected into 96 size fractions of 0.5 mm each and proteins were eluted into 96 well plates with elution buffer (8 M urea, 1% Triton-X100 in 100 mM Tris-HCl pH 9.5). Eluted proteins from each molecular weight fraction were loaded onto one distinct color of neutravidin-coated MagPlex beads (Luminex, Oosterhout, The Netherlands) and beads were pooled afterwards.

Aliquots of DigiWest bead mixes were transferred to 96 well plates containing assay buffer (blocking reagent for ELISA (Roche Applied Science, Mannheim, Germany) supplemented with 0.2% milk powder, 0.05% Tween-20 and 0.02% sodium azide). After discarding the assay buffer, diluted primary antibodies were added. Following overnight incubation on a shaker, bead mixes were washed with PBST, and phycoerythrin-labelled diluted secondary antibodies were added. Beads were washed twice, and assays were analyzed on a Luminex FlexMAP 3D device. A total of 103 different antibodies covering 93 targets plus additional controls were used.

Antibody-specific signals were quantified to identify peaks of correct molecular weight and to calculate peak areas using the DigiWest data analysis tool as described^42^. For comparative analyses between samples, protein expression values were normalized to beta-actin.

For hierarchical clustering (HCL) analysis the software package Multiexperiment Viewer MeV 4.8.1 was used^62^. The generated signals were normalized to the appropriate untreated controls and log2-transformed and sorted according to the four specified pathways. Each pathway’s list was then clustered on the antibody tree using Euclidian distance and complete linkage settings. Targets attributed to several pathways were clustered in each pathway list.

### Proliferation and colony forming unit (CFU) assays

To test proliferation, 100 cells per cm^2^ were seeded and cultured in the three different pHPL- media as described before. Medium was exchanged at day 2 and day 5. Total cell numbers were determined using C-Chip counting chambers (Merck) after 7 days. Delta (Δ) proliferation values of fibrinogen-depleted culture conditions were calculated from the ratio of the total cell numbers in +fib/+hep and fibrinogen depleted conditions. Cumulative population doublings were calculated as described previously^39^.

To prove if an overwhelming effect of pHPL derived growth factors on cell proliferation is masking a putative stimulating effect of heparin, stromal cell proliferation of different sources (n = 1 each) was tested with 10%, 3.3% and 1.1% pHPL adding 2 and 4 IU heparin/mL medium.

To investigate colony forming capacity, 1 cell per cm^2^ was seeded in cell culture dishes and cultured for 14 days. Medium was exchanged every third day. Colonies were fixed in 4% PFA (Sigma Aldrich) and stained with 0.05% Crystal Violet (Sigma Aldrich). Colonies defined as >50 cells were counted visually. Each assay was done in triplicate over three (proliferation, p1-3) or four (CFU assay, p1-4) subsequent passages.

### Real time monitoring of cell proliferation with unlabeled and labeled heparin

To exclude a distinct influence of fluoresceinamine-labeled (F-) heparin on cell proliferation, BM-, UC- and WAT-STCs were cultured in the presence of unlabeled and F-heparin. Experiments were performed by xCELLigence RTCA DP instrument (Roche Diagnostics) placed in a humidified incubator at 37 °C and ambient air conditions. After an incubation of cell-free growth medium for 30 minutes at room temperature, the background impedance was determined. For each measurement, 1 × 10^3^ cells per well were seeded in the presence of either 2 IU/mL standard heparin (Biochrom) or F-heparin (PG Research) into 16-well E-plates (Roche Diagnostics) in quadruplicates. To allow cell attachment, E-plates were stored at room temperature for 30 minutes. The E-plates were then locked in the RTCA DP device, the impedance value of each well was automatically monitored by the xCELLigence system and expressed as cell index (CI) value. Cells were cultured for 96–98 hours with CI monitoring every 5 minutes.

### *In vitro* tri-lineage differentiation assays

Adipogenic, and osteogenic differentiation potential of BM-, UC- and WAT-STCs (n = 3 each) was tested after expansion in the particular medium. For osteogenic and adipogenic differentiation, 1 × 10^3^ cells per cm^2^ (passage 2) were seeded. After 24 hours, medium was replaced by differentiation medium as described^5^. At day 14, cells were stained after fixation with 4% paraformaldehyd (PFA, Sigma Aldrich) either with 0.5% Alizarin Red (Sigma Aldrich) or 1% Sudan III (Sigma Aldrich). Photographs were taken using a PrimoVert Light microscope and an AxioCam ERc5s digital camera (both Zeiss, Germany).

*In vitro* 3D chondrogenic differentiation of BM-STCs (n = 3) was performed as previously published^5,63^. Briefly, 5 × 10^5^ cells were seeded onto collagen I-coated (Sigma Aldrich) transwells (Corning, Corning, NY, USA). Cartilage discs were grown in chondrogenic differentiation medium consisting of Dulbecco’s Modified Eagle Medium (DMEM) high glucose (Sigma Aldrich) supplemented with 40 µg/mL L-prolin (Sigma Aldrich), 10^−7^ M dexamethasone (Stem cell technologies, Vancouver, Canada), 25 µg/mL L-ascorbic acid 2-phosphate (Sigma Aldrich), 1x Insulin-transferrinsodium selenite plus linoleic-BSA (ITS + 1) cell culture supplement (Sigma Aldrich), 1x sodium pyruvate (Sigma Aldrich), 1x L-glutamine (Life Technologies), 1x Pen/Strep (Sigma Aldrich) and 10 ng/mL TGFbeta (Pepro Tech, London, UK). Cultures were incubated at 37 °C with medium being changed every second day. After four weeks, the cartilage discs were harvested and weights were measured. Subsequently, discs were formalin-fixed, paraffin-embedded and processed into 4 μm sections. Briefly, after deparaffinizing and hydrating the paraffin sections, they were incubated in Weigert’s Iron Hematoxylin (Hematoxylin, Ferric Chloride, both Merck, Darmstadt, Germany) for 5 minutes and afterwards washed in distilled water for three times. The sections were then differentiated in 1% acid-alcohol for 10 seconds and rinsed in distilled water three times followed by an incubation for 1 minute in 0.2% Fast Green (Morphisto, Frankfurt, Germany), 15 seconds in 1.0% acetic acid and 30 minutes in 1.0% Safranin O (Merck). Slides were briefly rinsed in 96% ethanol and dehydrated with two changes of 96% ethanol and 100% ethanol. Finally, the sections were washed in acetic acid n-butyl ester (Carl Roth, Karlsruhe, Germany) and mounted. Stained paraffin sections on slides were automatically scanned in 40x magnification using the Olympus slidescanner VS120 and the Olympus VS-ASW-L100 program (both Olympus, Tokyo, Japan). The Bern Score evaluation was done by three independent observers as previously published by evaluating the SafraninO/Fast Green stained paraffin sections^47^.

### Statistical analysis for data other than microarray data

Data are presented as mean ± SD. D’Agostino and Pearson omnibus normality test were applied to test for Gaussian distribution. Data were compared using 2-way ANOVA and Tukey’s range test or unpaired t-Test. Analysis was done using GraphPad Prism 7 (GraphPad Software, La Jolla, CA, USA) and p ≤ 0.05 was considered as significant (indicated by asterisk).

## Supplementary information

Supplementary Figures and Tables

## Data Availability

The datasets generated during and/or analyzed during the current study are available from the corresponding author on reasonable request.
