# Supplementary material for: Heparin Differentially Impacts Gene Expression of Stromal Cells from Various Tissues
Source: Sci Rep. 2019 May 10;9:7258. doi: 10.1038/s41598-019-43700-x (PMC6510770; doi:10.1038/s41598-019-43700-x)
Supplement: Supplementary file 1 — Supplementary Figures and Tables [file 41598_2019_43700_MOESM1_ESM.pdf]

SUPPLEMENTARY MATERIAL

Heparin Differentially Impacts Gene Expression of Stromal Cells from Various Tissues

Sandra Laner-Plamberger[1,2], Michaela Oeller[1,2], Rodolphe Poupardin[1,3], Linda Krisch[1,2], Sarah Hochmann[1,3], Ravi Kalathur[1,3,6], Karin Pachler[1,4], Christina Kreutzer[1,5], Gerrit Erdmann[7], Eva Rohde[1,2], Dirk Strunk[1,3,#], and Katharina Schallmoser[1,2,#,*]

[1] Spinal Cord Injury and Tissue Regeneration Center Salzburg, [2] Department of Transfusion Medicine, [3] Cell Therapy Institute, [4] GMP Unit, [5] Institute for Experimental Neuroregeneration, Paracelsus Medical University of Salzburg, Austria

[6] Department for Biomedicine, University of Basel, Switzerland

[7] NMI TT Pharmaservices, Berlin, Germany

[#] DS and KS contributed equally to this manuscript.

* Corresponding author:

Prof. Dr. Katharina Schallmoser

Department of Transfusion Medicine

Spinal Cord Injury & Tissue Regeneration Center Salzburg (SCI-TReCS)

Paracelsus Medical University of Salzburg

Strubergasse 21, A - 5020 Salzburg, Austria

E-Mail: k.schallmoser@salk.at

Tel: +43 5 7255-57987

Fax: +43 5 7255-24599

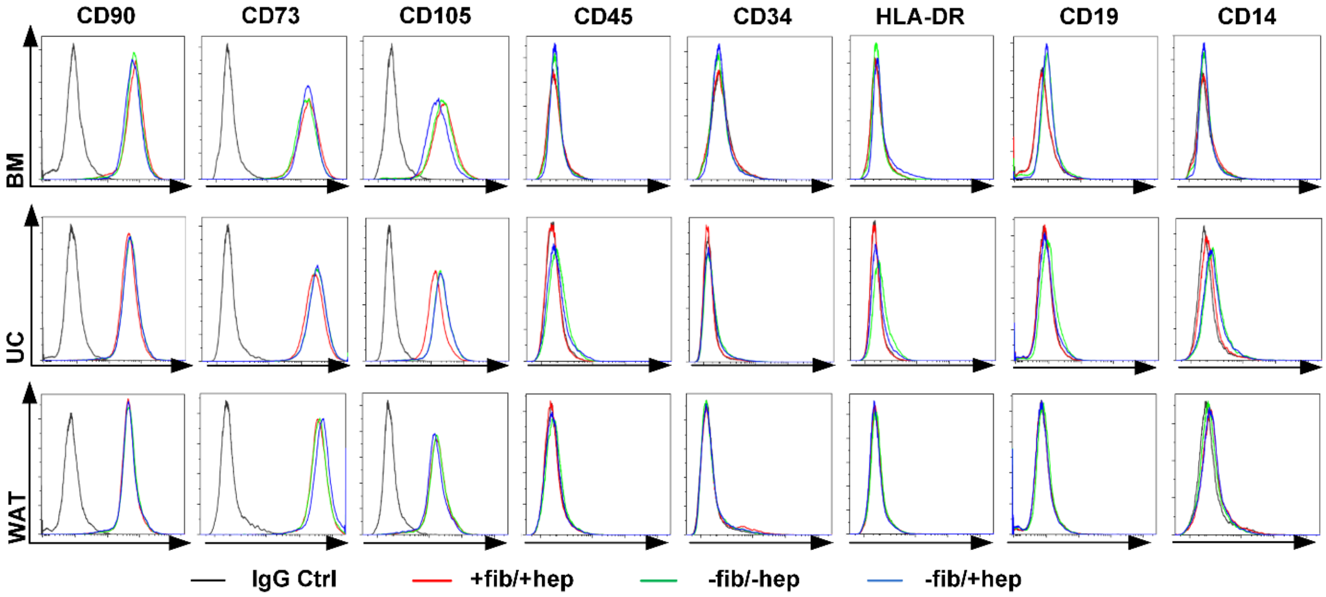

Figure S1: Immunophenotyping of stromal cells by flow cytometry*. Histogram plots show overlay results of surface marker expression of one representative donor for each cell source (bone marrow BM, umbilical cord UC or white adipose tissue WAT) cultured either in the presence of fibrinogen and heparin (+fib/+hep, red), in the absence of both (-fib/-hep, green) or in the absence of fibrinogen and presence of heparin (-fib/+hep, blue). IgG controls are indicated in black.*

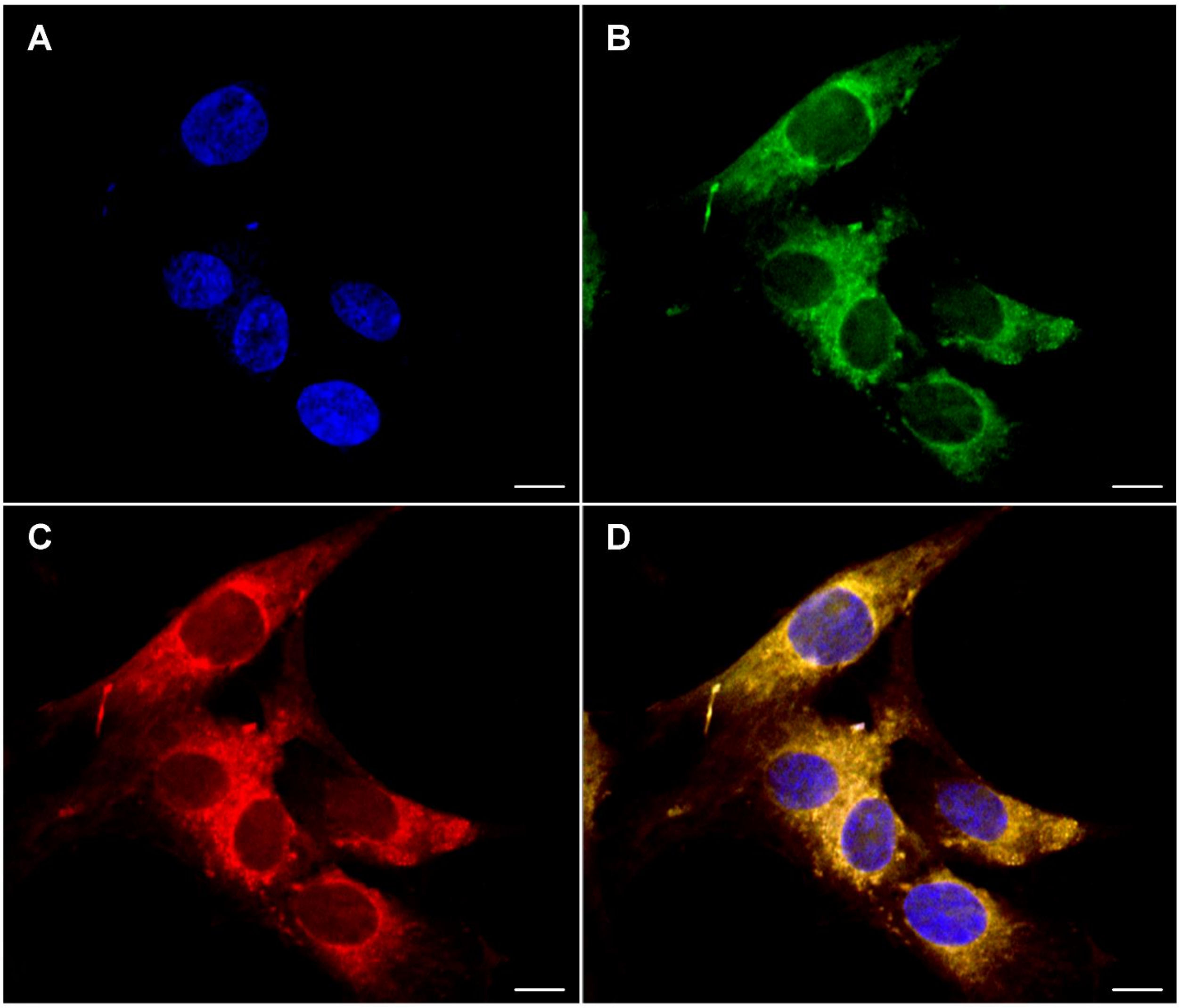

Figure S2A: F-heparin was internalized in lysosomes of stromal cells.

*Z-stack immunofluorescence images from one representative donor of UC-derived stromal cells treated simultaneously with F-Heparin (green) and LysoTracker (red) indicate the localization of internalized F-heparin in lysosomes. (**A**) DAPI staining, (**B**) F-Heparin (**C**) LysoTracker and (**D**) overlay of **A**-**C**. Scale bars = 10 µm.*

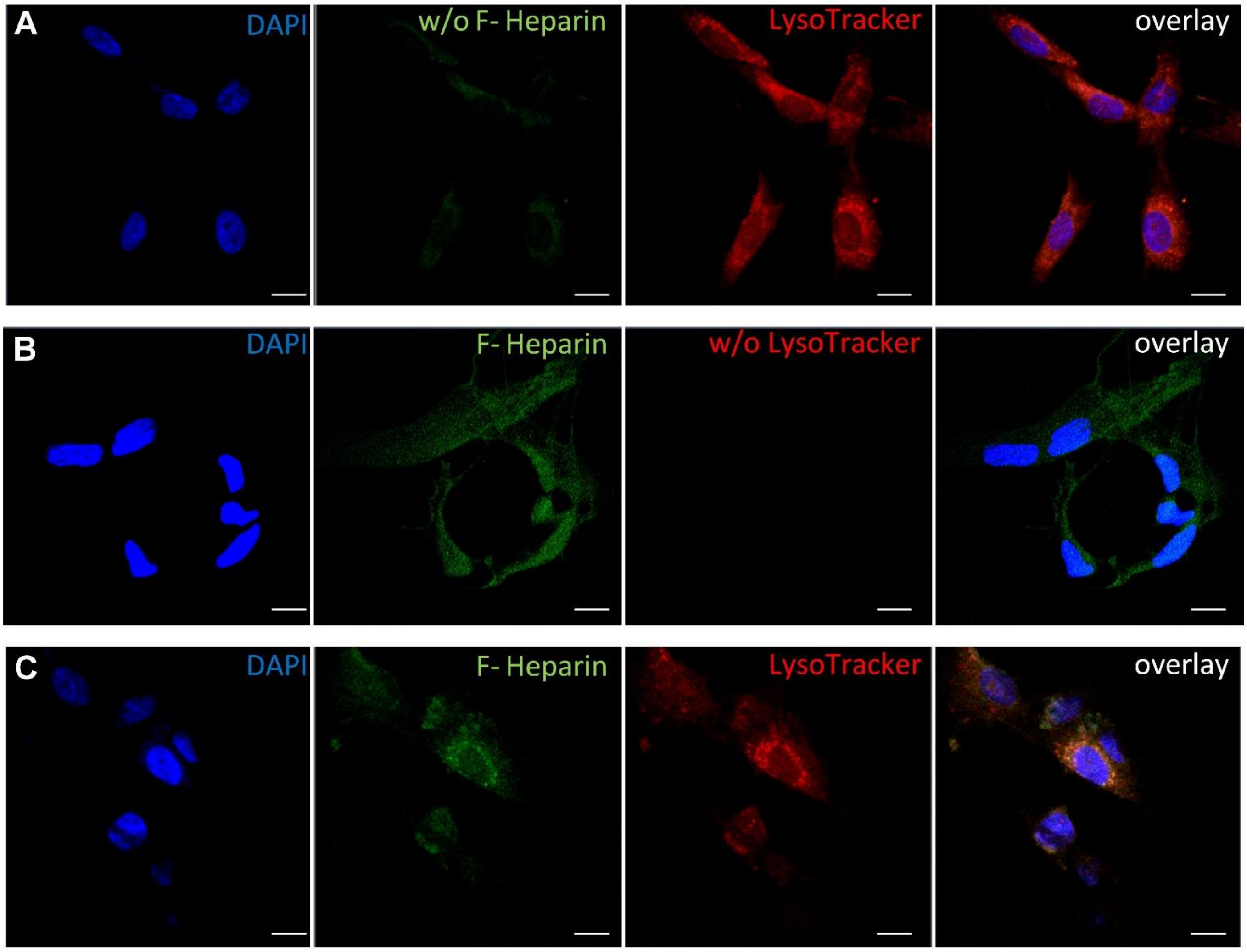

***Figure S2B**: Z-stack immunofluorescence showing (**A**) staining with DAPI (blue) and LysoTracker (red), but without (w/o) F-Heparin, **(B)** staining with DAPI and F-Heparin (middle panel) without LysoTracker and **(C)** simultaneous staining of DAPI, F-Heparin and LysoTracker from one representative donor. Overlays of stainings are shown on the right side. Scale bar = 10 µm.*

WNT pathway – 1/4

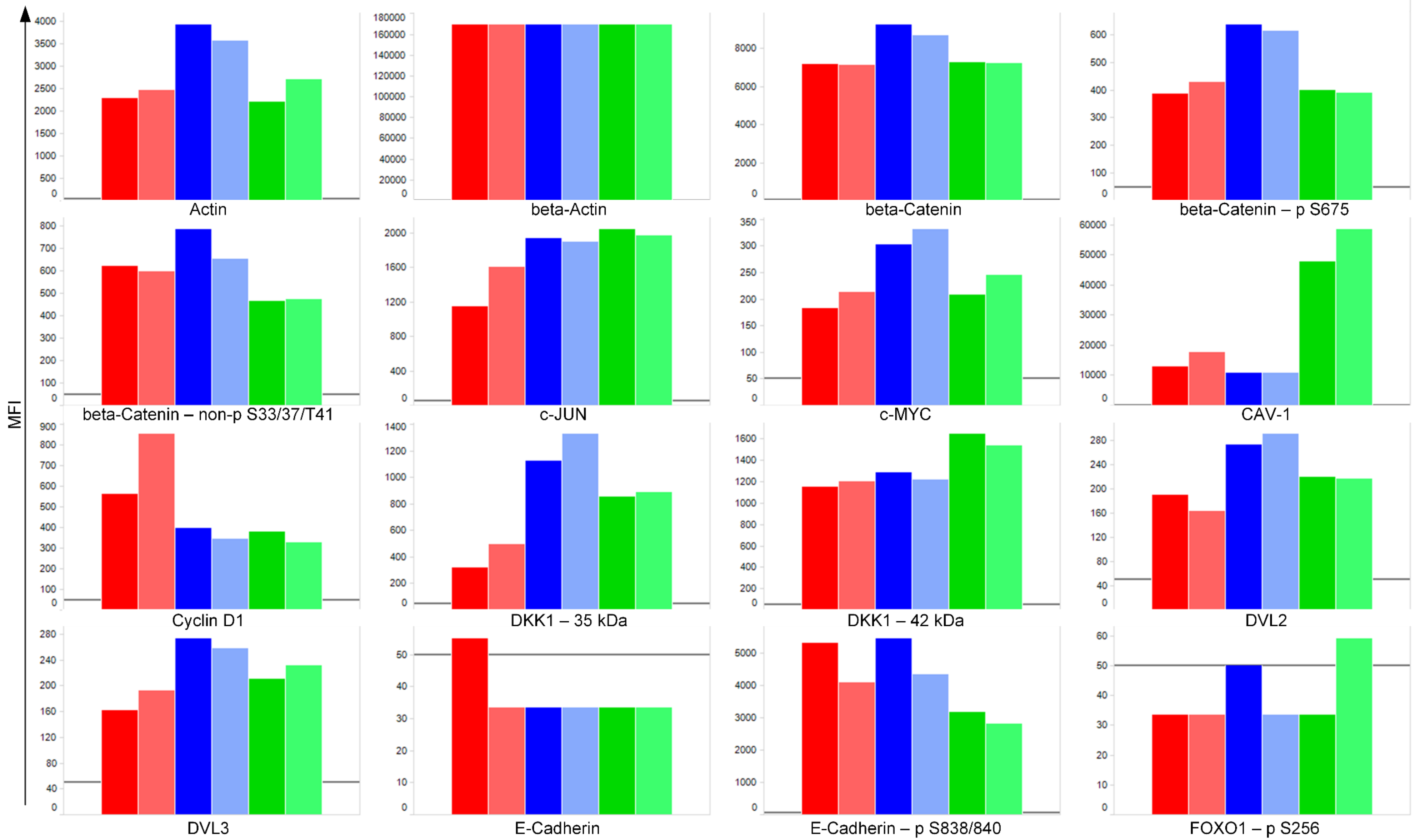

WNT pathway – 2/4

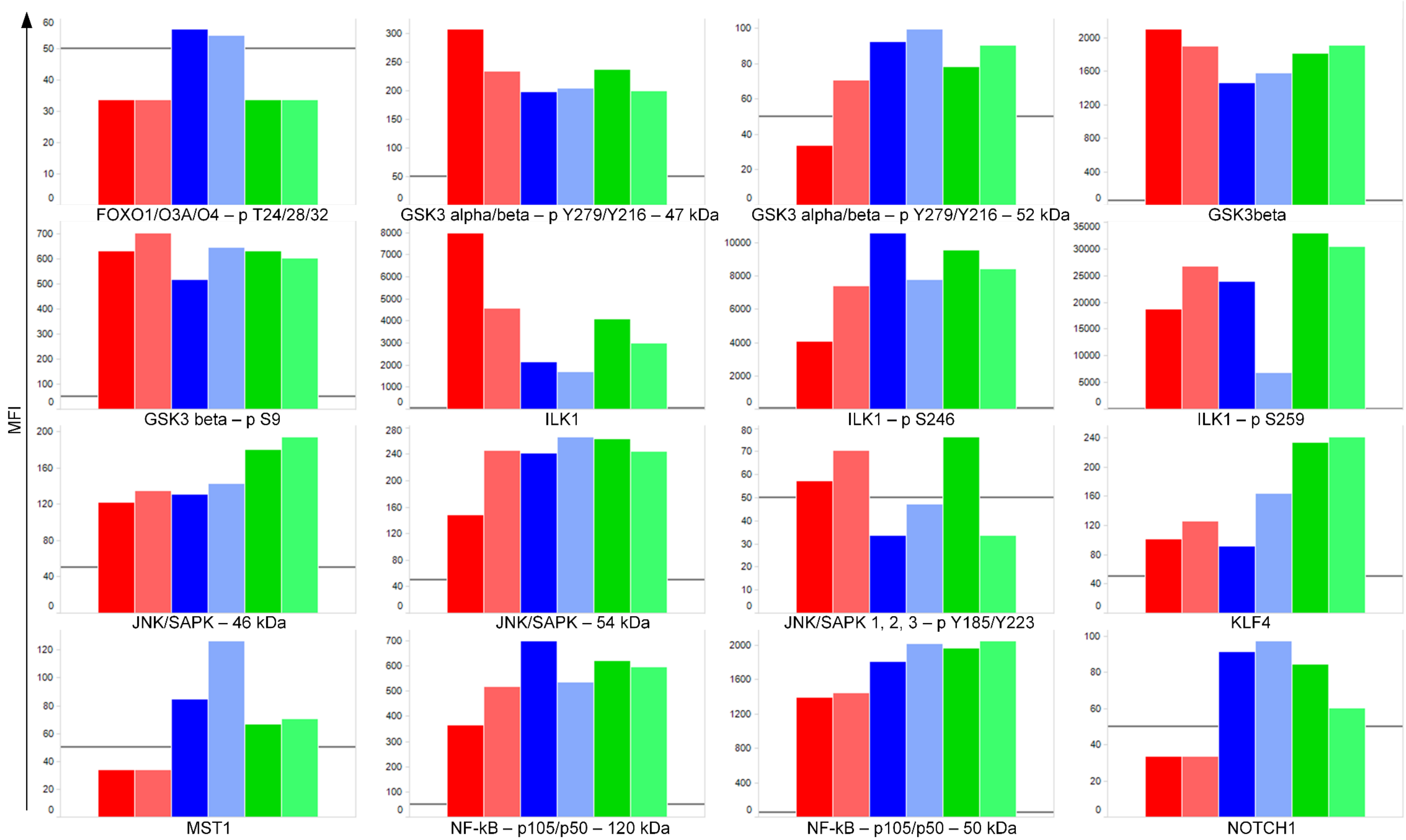

WNT pathway – 3/4

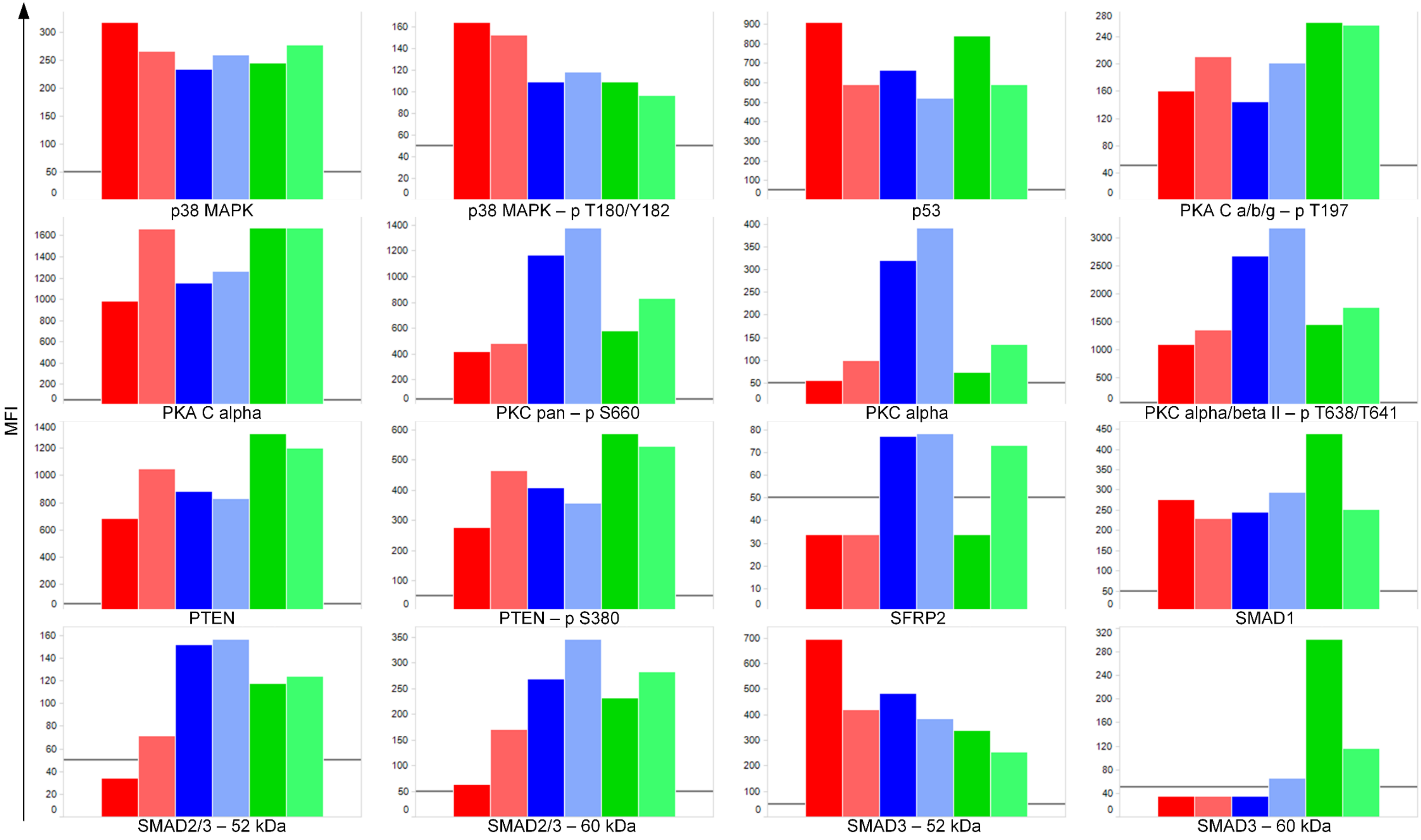

WNT pathway – 4/4

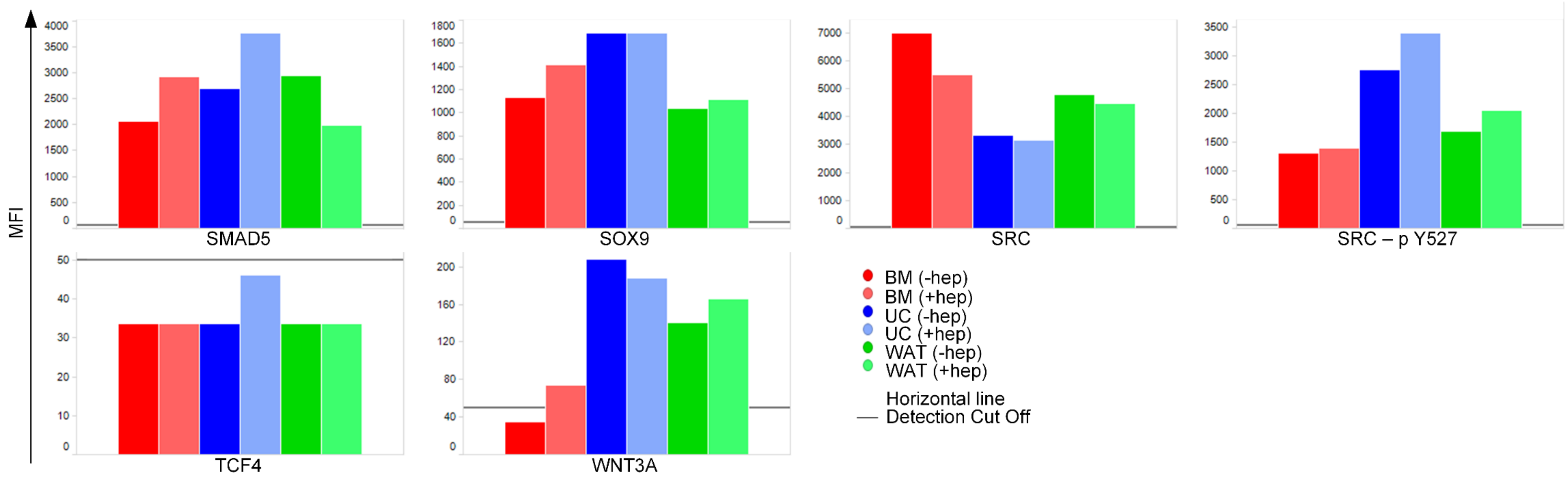

PDGF pathway – 1/3

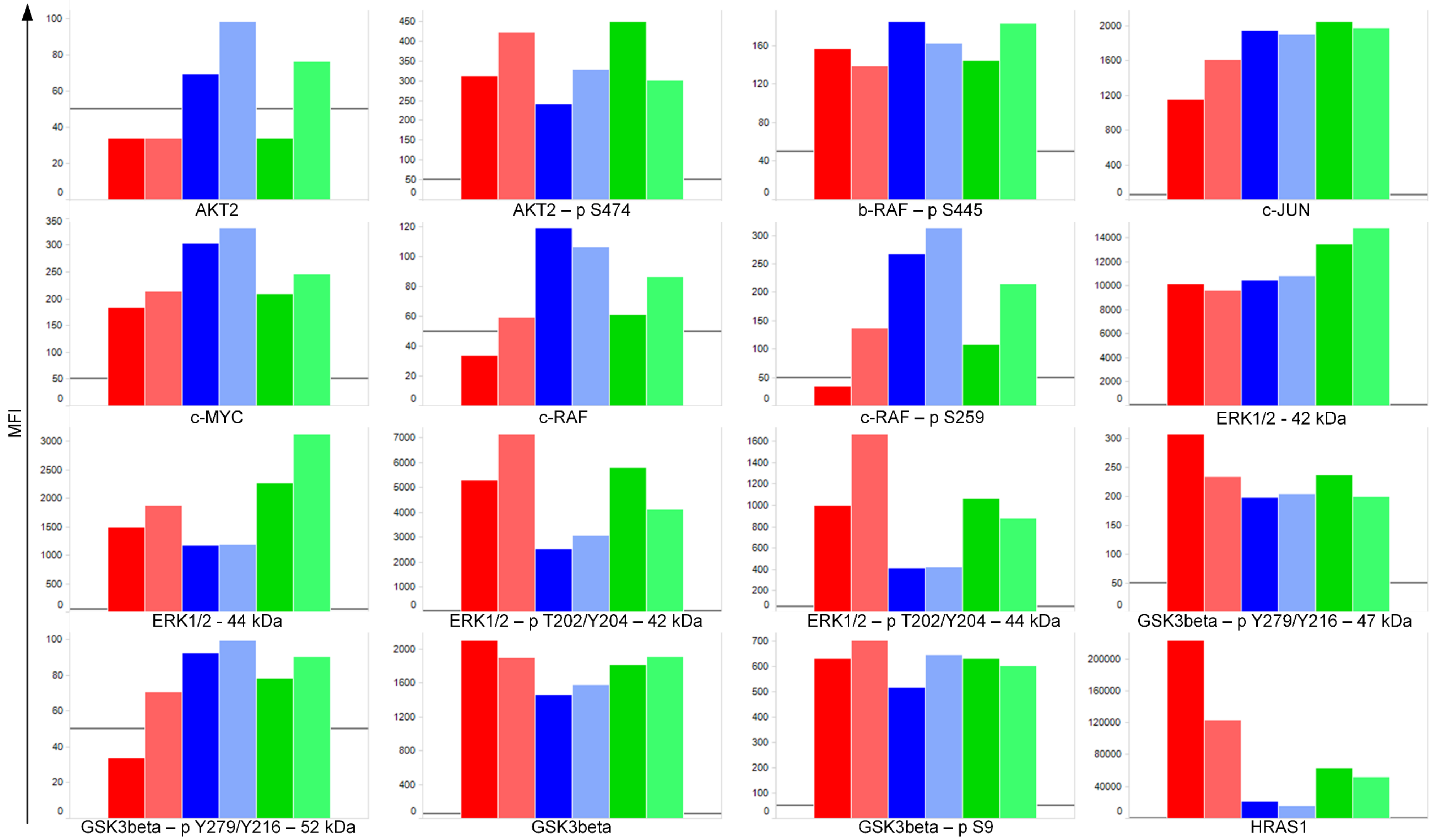

PDGF pathway – 2/3

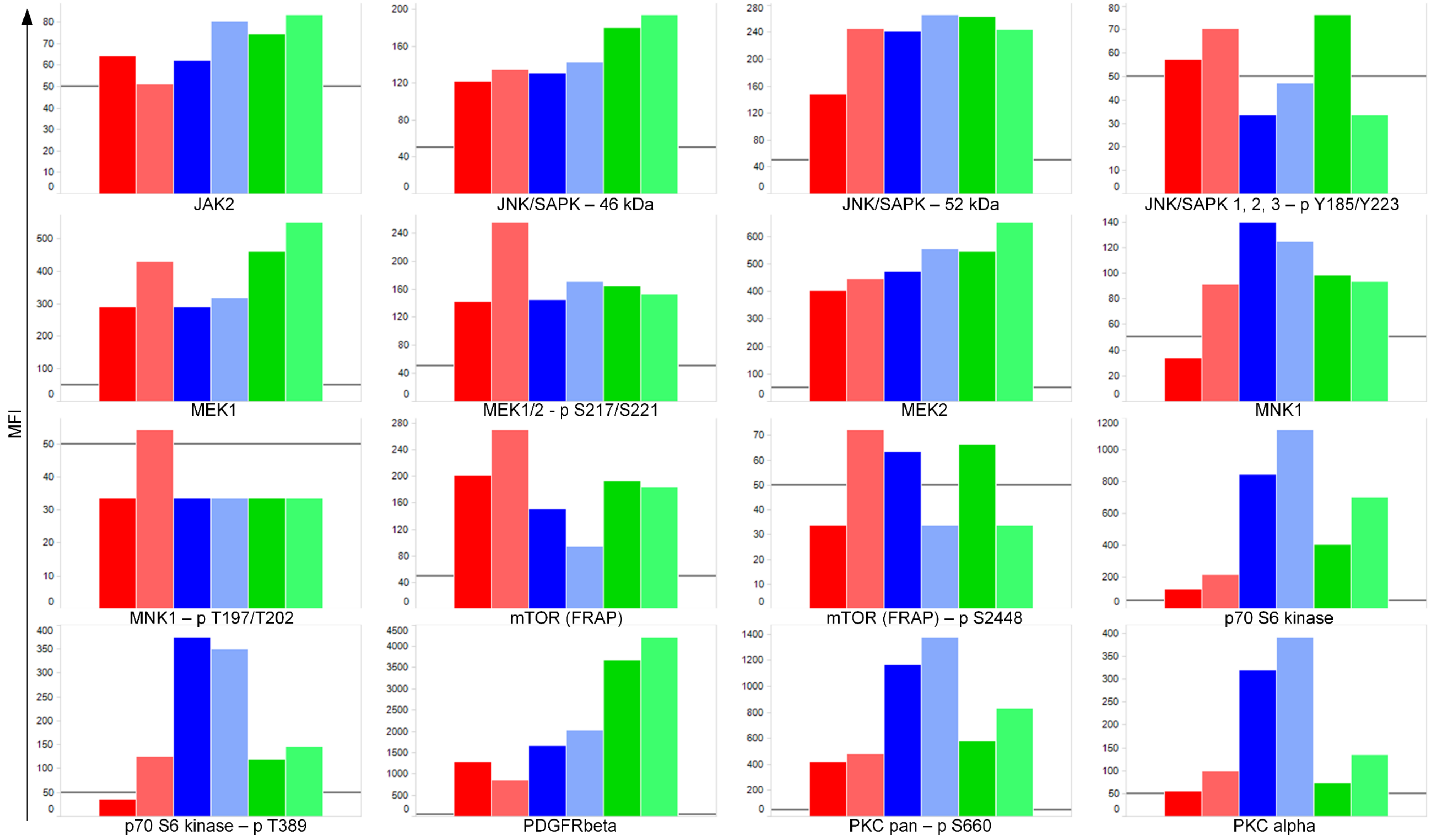

PDGF pathway – 3/3

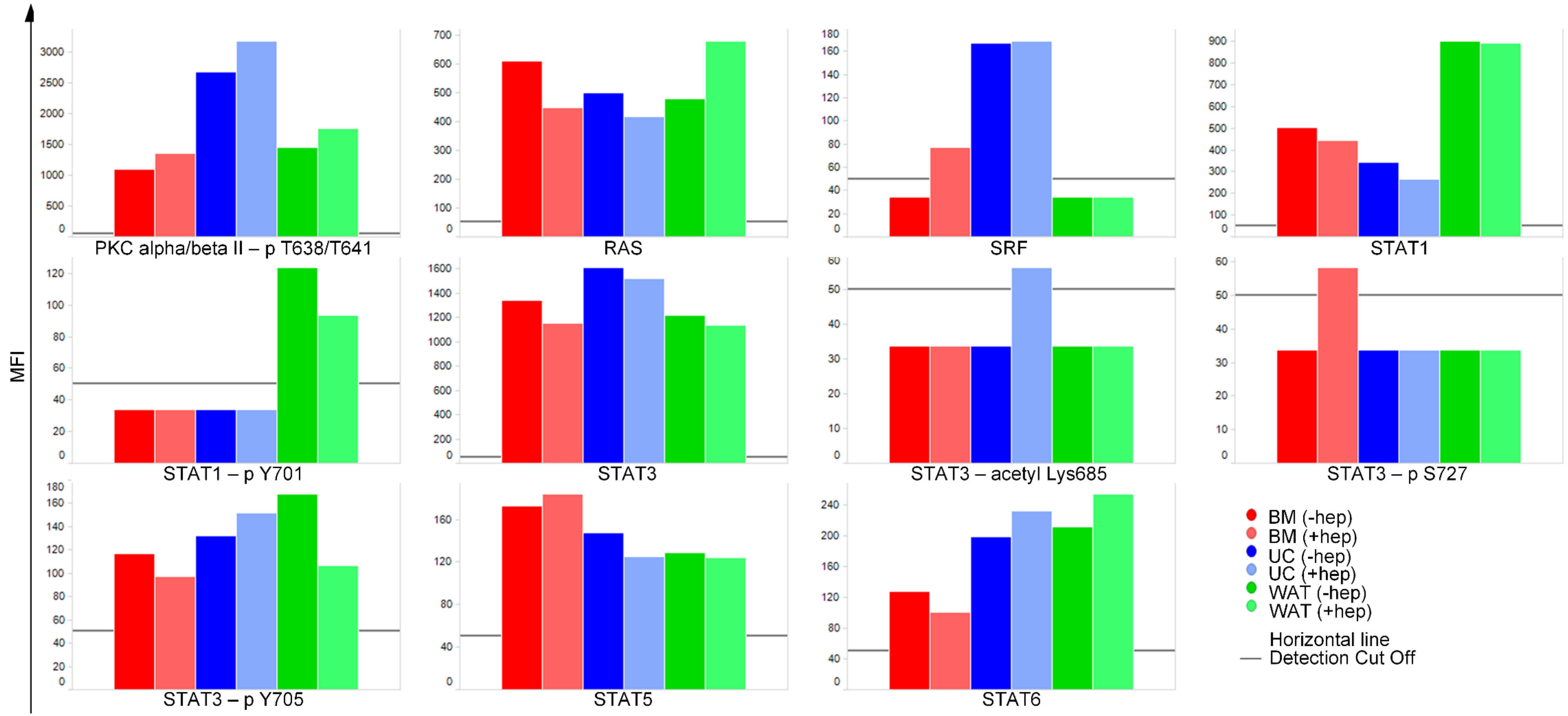

83

TGFbeta pathway – 1/2

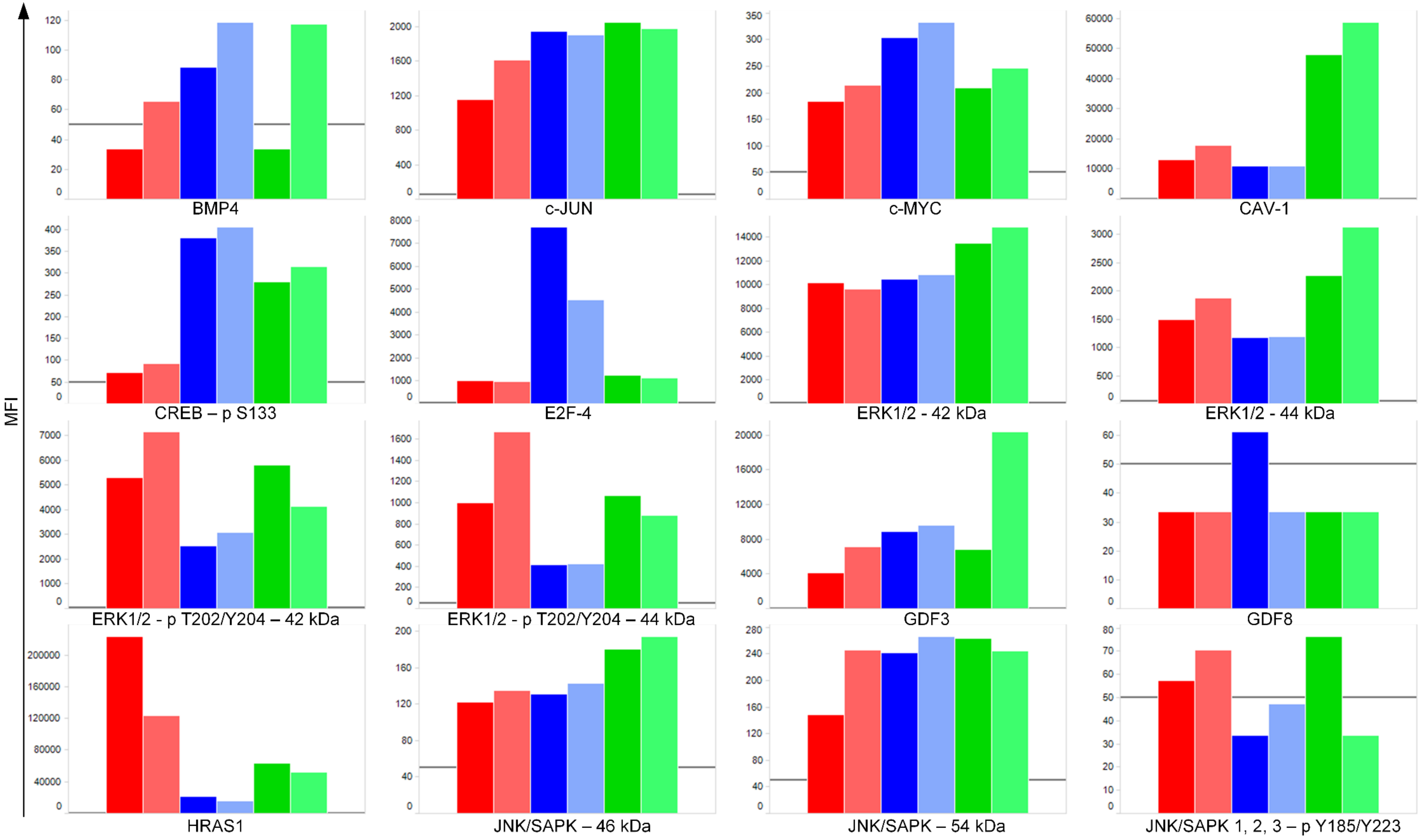

84
85

86

TGFbeta pathway – 2/2

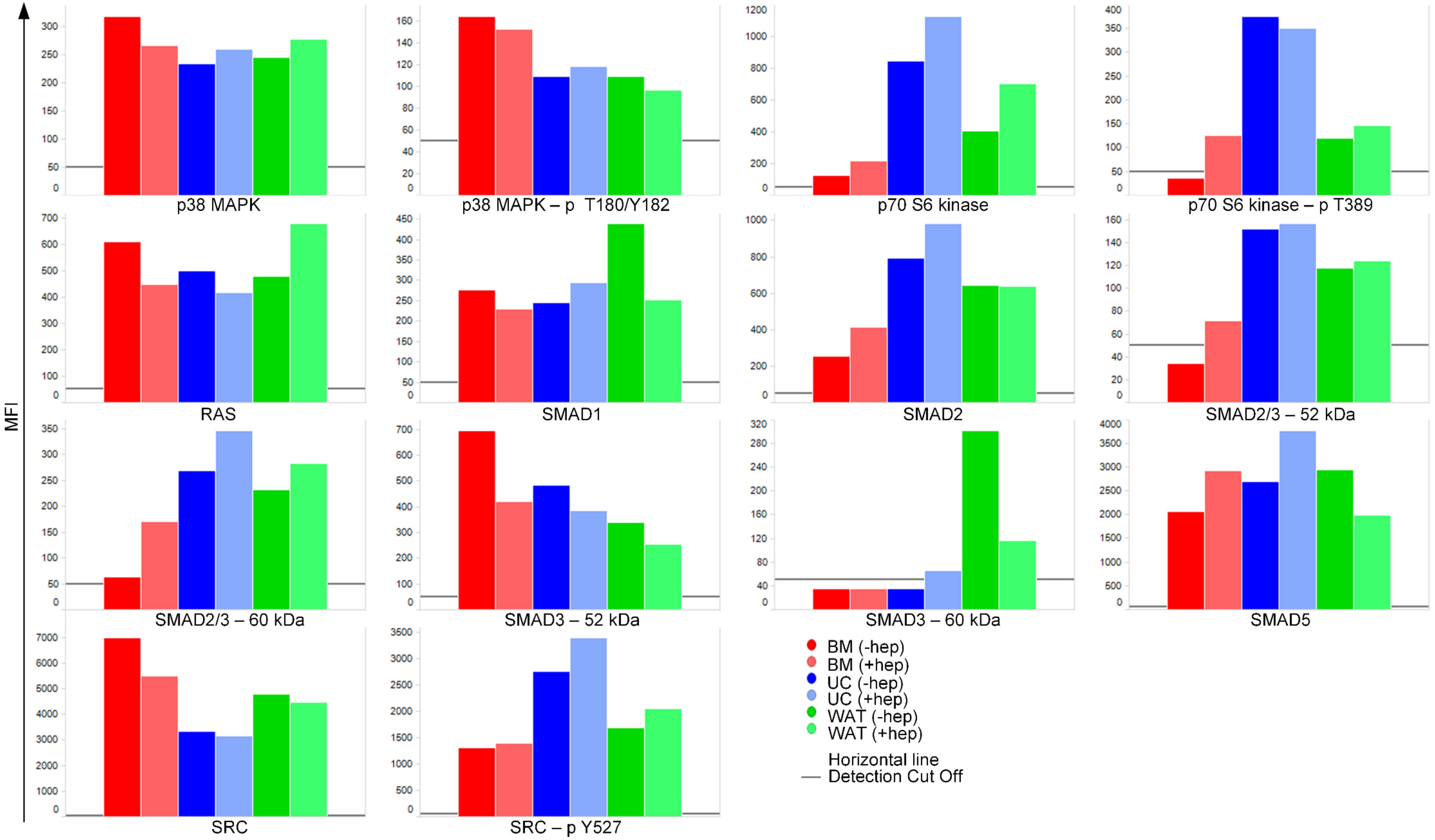

87

NOTCH pathway

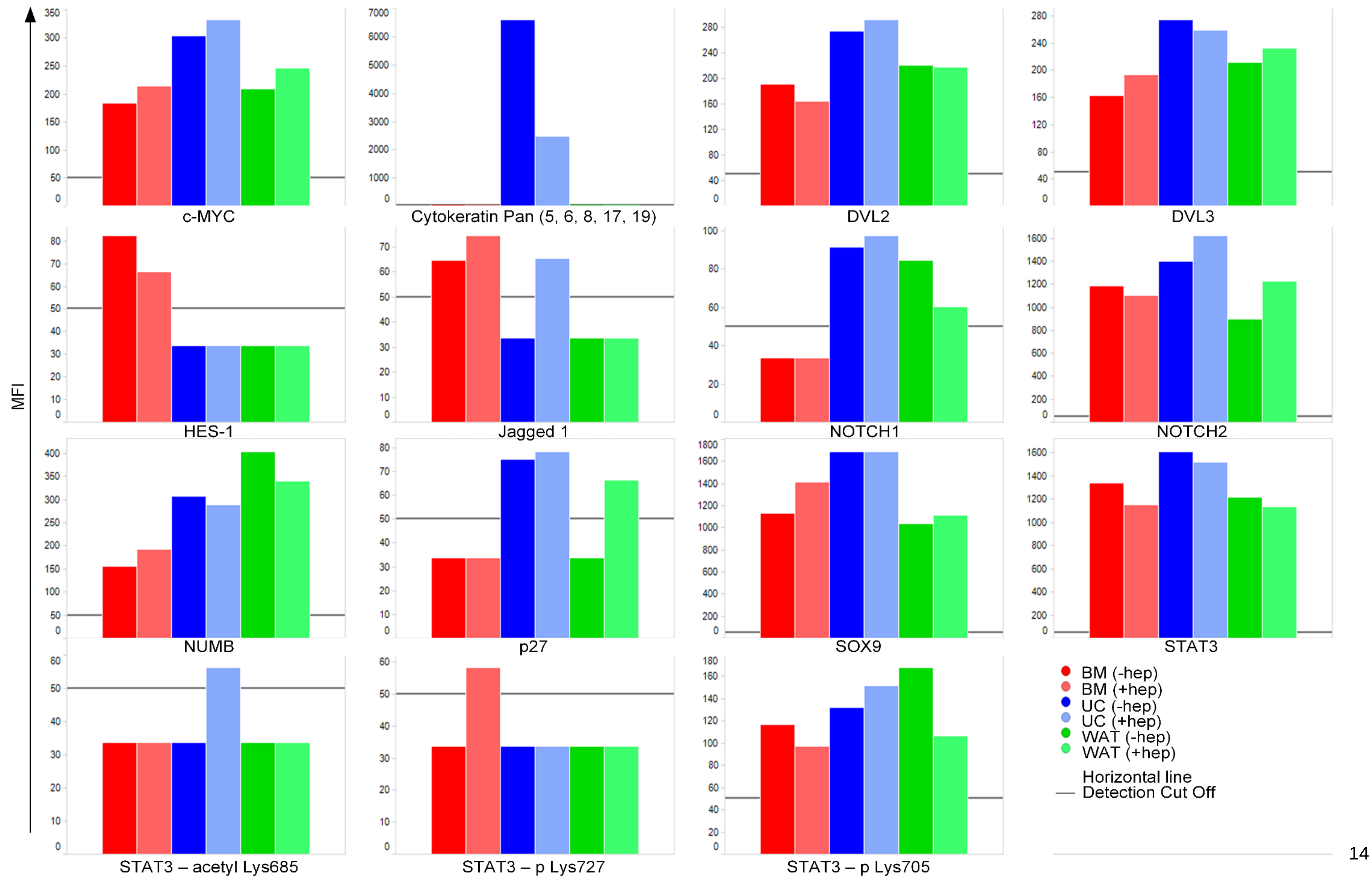

***Supplementary Figure S3: Heparin affects a distinct protein expression of WNT-, PDGF-, TGFbeta- and NOTCH signaling pathways**. Bead-based western blotting (DigiWest) of BM- (red), UC- (blue) and WAT-derived (green) stromal cells cultivated either in the absence (-hep) or presence of heparin (+hep). All 97 tested analytes are sorted according to the four pathways. Data are shown as mean fluorescence intensity (MFI) values of one donor per source (hep = heparin).*

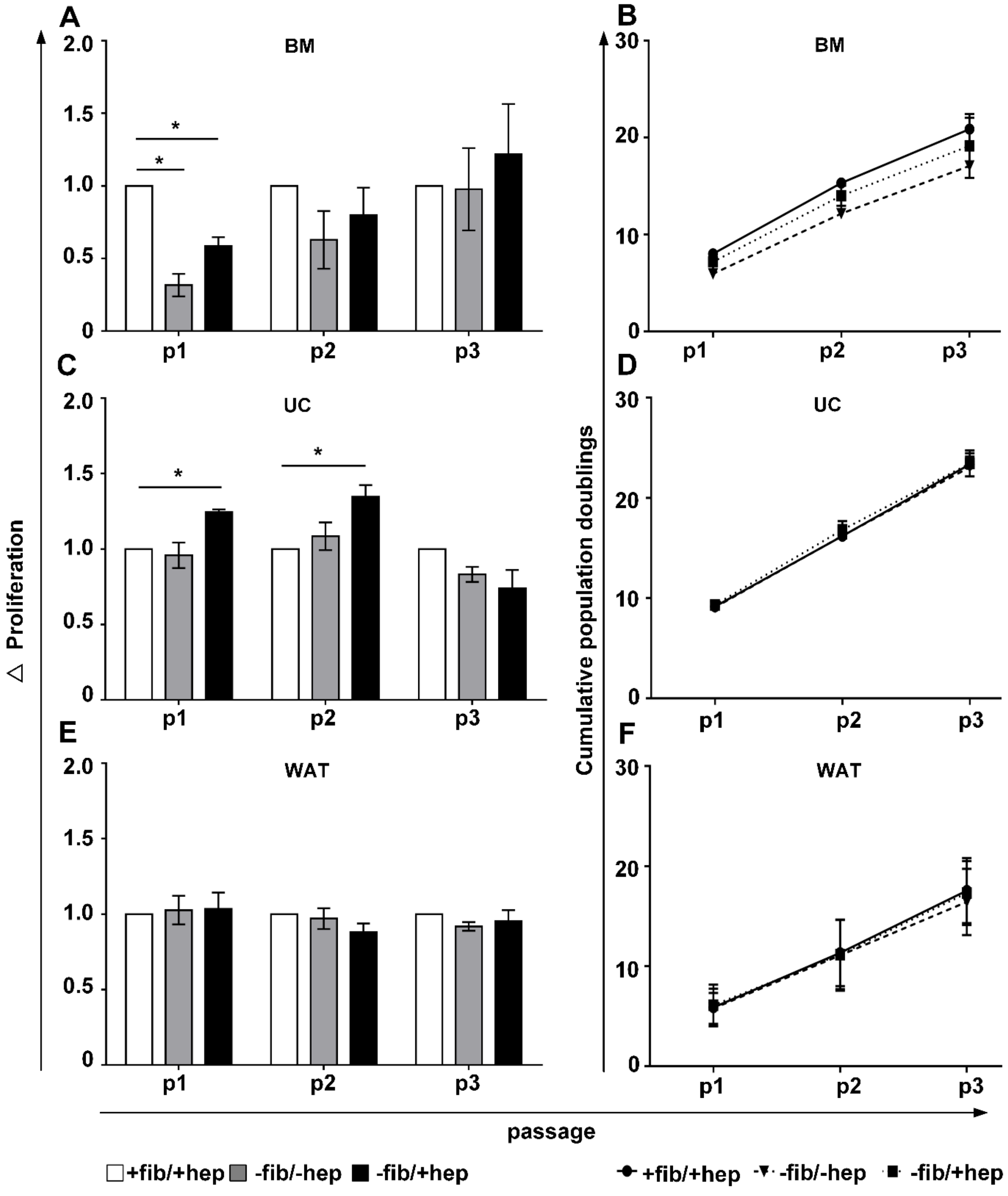

Figure S4: Proliferation rate of BM-, UC- and WAT stromal cells in the presence and absence of heparin. *Proliferative capacity of stromal cells derived from BM, UC and WAT was determined over three passages (p1-p3) in fibrinogen-depleted pHPL-medium (grey bars, -fib/-*

*hep) or in fibrinogen-depleted pHPL-medium containing heparin (black bars, -fib/+hep) compared to standard pHPL-medium (white bars, +fib/+hep) for (**A**) BM stromal cells, (**C**) UC stromal cells and (**E**) WAT stromal cells. Data are shown as △ proliferation rates of three independent donations for each tissue source measured in duplicates (mean ± SD, * $p < 0.05$, fib = fibrinogen, hep = heparin). Cumulative population doublings (p1-p3) are shown for (**B**) BM-, (**D**) UC- and (**F**) WAT stromal cells.*

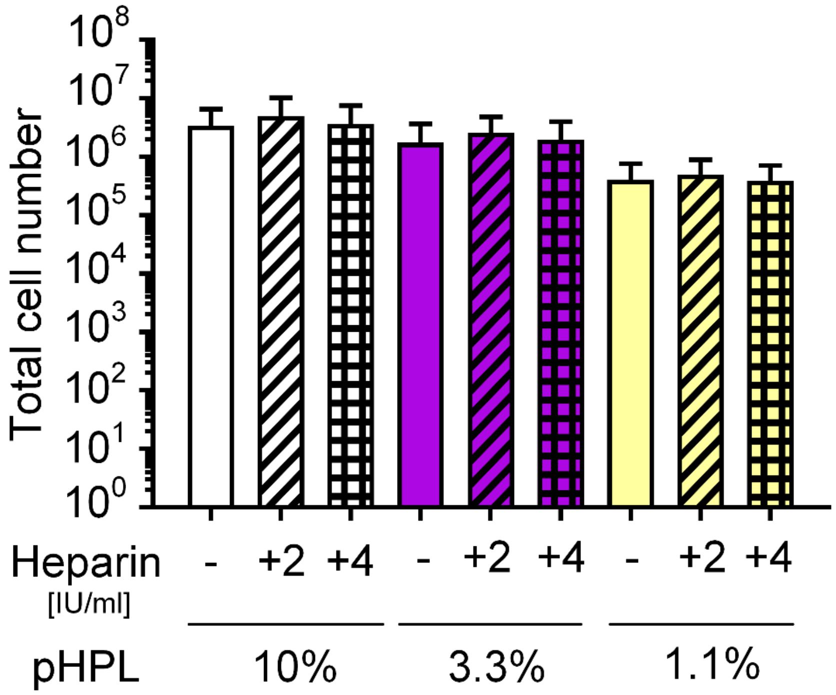

Figure S5: Titration of pHPL to reduce growth factor concentrations. *To show whether the growth factors in 10% fibrinogen depleted pHPL may cover an effect of heparin on cell proliferation, we reduced the pHPL concentration in the cell culture medium by titration (from 10% to 3.3% and 1.1%). Furthermore, the effect of adding 2 and 4 IU/mL heparin on cell proliferation of stromal cells was investigated. Total numbers of stromal cells are shown as mean ± SD.*

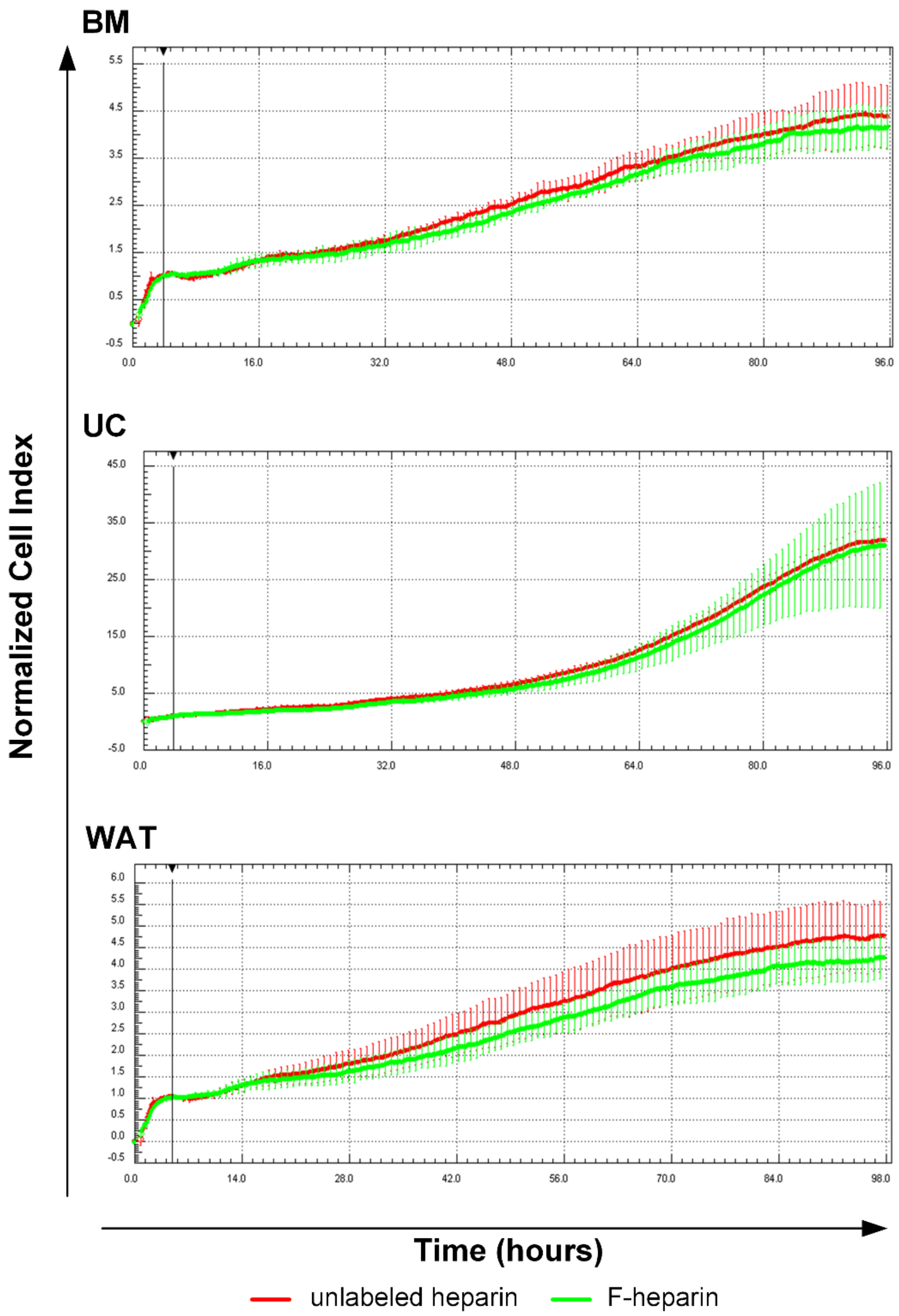

Figure S6: Effect of heparin and F-heparin on stromal cell proliferation. *Using XCELLigence impedance measurement, the proliferative behavior of BM-, UC- and WAT stromal cells exposed to unlabeled heparin (red) and fluoresceinamine-labeled heparin (F-heparin, green) was analyzed.*

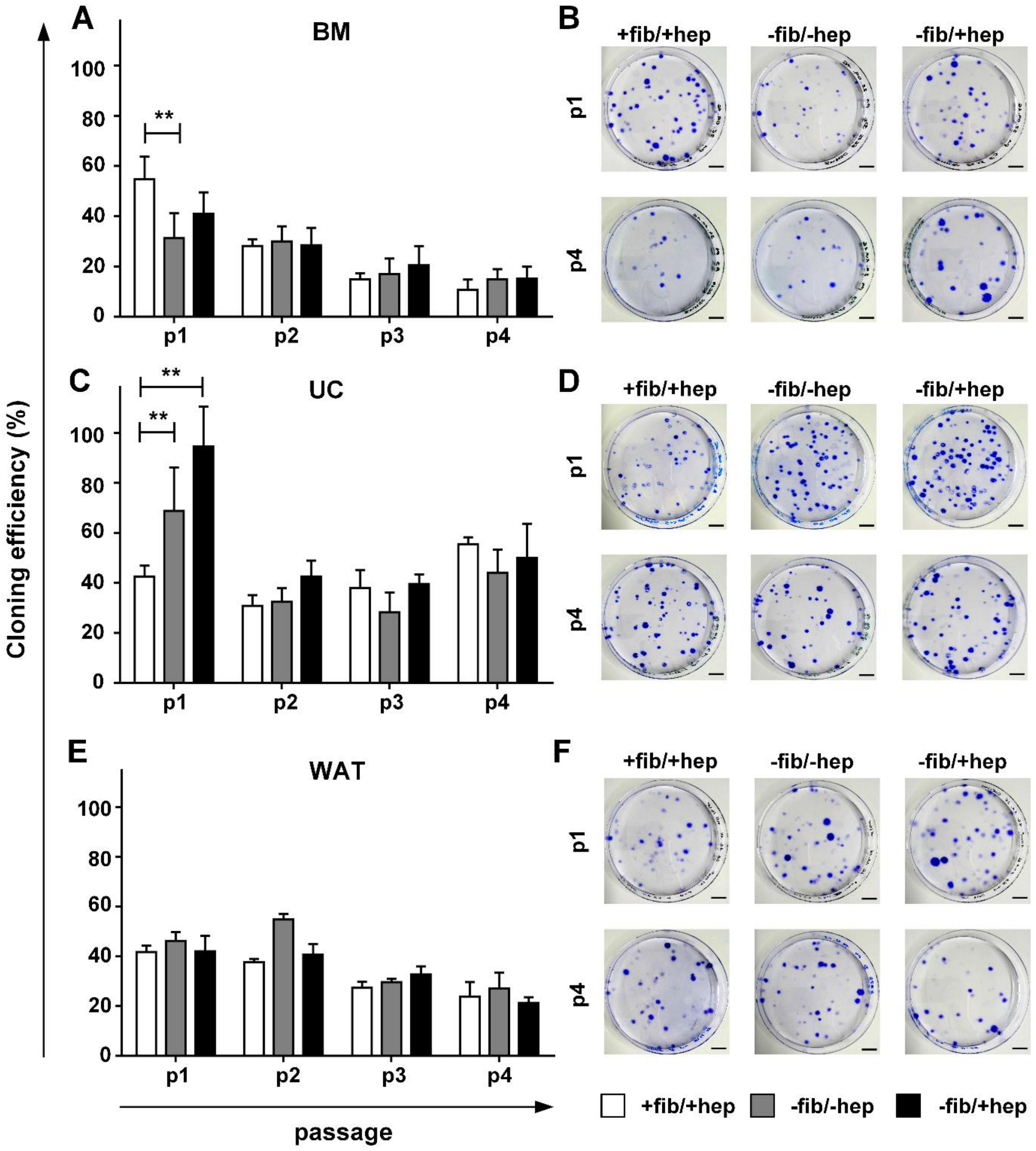

Figure S7: Clonogenicity of stromal cells. *The cloning efficiency (%) over four passages in different pHPL-media of stromal cells isolated from (**A**, **B**) BM, (**C, D**) UC or (**E, F**) WAT, (n = 3) is shown (mean ± SD of triplicates; ** p < 0.01). (**B, D, F**) Photographs of colony forming units from one representative donor of each tissue source cultured in medium types as indicated (100 mm cell culture dishes) are shown for passages one and four (fib = fibrinogen, hep = heparin).*

	#	Gene name		Function according to Entrez Gene Summary	FC
BM	1	***NIPSNAP3B***	Nipsnap Homolog 3B	Protein coding gene; putative role in vesicular trafficking	**2.76 ****
	2	***PDGFD***	Platelet Derived Growth Factor D	Member of the platelet-derived growth factor family; mitogenic factor with essential role in the regulation of embryonic development, cell proliferation, cell migration, survival and chemotaxis	**2.57 ***
	3	***CD74***	CD74 Molecule, invariant chain	Chaperone and cell surface receptor; associates with class II major histocompatibility complex (MHC); serves as cell surface receptor for the cytokine macrophage migration inhibitory factor (MIF) which, when bound to the encoded protein, initiates survival pathways and cell proliferation	**2.44 ****
	4	***CFI***	Complement Factor I	Serine proteinase, regulates complement cascade	**2.39 ***
	5	***SVEP1***	Sushi, Von Willebrand Factor Type A, EGF And Pentraxin Domain Containing 1	May play a role in the cell attachment process	**2.34 ***
	6	***RANBP3L***	RAN Binding Protein 3 Like	Nuclear export factor for SMADs, critical role in terminating BMP signaling and regulating mesenchymal stem cell differentiation	**2.20 ***
	7	***ANO3***	Anoctamin 3	TMEM16 family member of predicted membrane proteins; may act as potassium channel regulator and inhibit pain signaling	**2.18 ***
	8	***ANGPT1***	Angiopoietin 1	Glycoprotein, important for regulation of angiogenesis, endothelial cell survival, proliferation, migration, adhesion and cell spreading, reorganization of the actin cytoskeleton, but also maintenance of vascular quiescence	**2.15 ****
	9	***SPRY1***	Sprouty RTK Signaling Antagonist 1	May function as an antagonist of fibroblast growth factor (FGF) pathways	**2.14 ***
	10	***PRB2***	Proline Rich Protein BstNI Subfamily 2	Basic, proline-rich, human salivary glycoprotein	**2.08 ****
	11	***ITGB8***	Integrin Subunit Beta 8	Single-pass type I membrane protein, receptor for fibronectin	**2.04 ***
	12	***GUCY1B3***	Guanylate Cyclase 1 Soluble Subunit Beta 1	Soluble guanylate cyclase, catalyzes biosynthesis of cGMP	**2.03 ***
	13	***JAM2***	Junctional Adhesion Molecule 2	Adhesive ligand for interacting with a variety of immune cell types, and may play a role in lymphocyte homing to secondary lymphoid organs	**1.98 ***
	14	***BMP4***	Bone Morphogenetic Protein 4	Ligand, binds to TGF receptors, leading to recruitment and activation of SMAD family transcription factors that regulate gene expression, induces cartilage and bone formation	**1.96 ***
	15	***SEPP1***	Selenoprotein P	Implicated as an extracellular antioxidant, and in the transport of selenium to extra-hepatic tissues	**1.95***
UC	1	***CEMIP***	Cell Migration Inducing Hyaluronan Binding Protein	Binds and hydrolyzes high molecular weight hyaluronic acid to produce an intermediate-sized product, related to glycosaminoglycan metabolism	**6.10 *****
	2	***WNT2***	Wnt Family Member 2	Member of the WNT gene family, ligand for frizzled receptors, developmental protein	**5.20 ****
	3	***COLEC12***	Collectin Subfamily Member 12	Member of the C-lectin family, scavenger receptor, a cell surface glycoprotein that displays several functions associated with host defense	**3.94 ***
	4	***OSR1***	Odd-Skipped Related Transcription Factor 1	Transcription factor that plays a role in the regulation of embryonic heart and urogenital development	**3.88 ****
	5	***VCAM1***	Vascular Cell Adhesion Molecule 1	Member of the Ig superfamily, cell surface sialoglycoprotein, Important in cell-cell recognition, mediates leukocyte-endothelial cell adhesion and signal transduction	**3.31 ***
	6	***SLC7A14***	Solute Carrier Family 7 Member 14	Amino acid transporter protein, transmembrane protein	**3.28 ***
	7	***FAM65C***	RIPOR Family Member 3	Protein coding gene	**3.03 ****
	8	***C3***	Complement C3	Central player of activation of complement system	**2.65 ***

	9	***IL34***	Interleukin 34	Cytokine that promotes the proliferation, survival and differentiation of monocytes and macrophages, promotes the release of pro-inflammatory chemokines, plays an important role in innate immunity and in inflammatory processes, important for regulation of osteoclast proliferation and differentiation	**2.58 ***
	10	***ITGB8***	Integrin Subunit Beta 8	Single-pass type I membrane protein, receptor for fibronectin	**2.57 ****
	11	***DACH1***	Dachshund Family Transcription Factor 1	Chromatin-associated protein, associates with other transcription factors to regulate gene expression and cell fate determination	**2.56 ***
	12	***TAS2R46***	Taste 2 Receptor Member 46	Belongs to the TAS2R receptor family, expressed on the surface of taste receptor cells and mediates the perception of bitterness	**2.47 ***
	13	***ENPP2***	Ectonucleotide Pyrophosphatase/Phosphodiesterase 2	Phosphodiesterase and phospholipase, acts as an angiogenic factor by stimulating the migration of smooth muscle cells, promotes microtubule formation	**2.46 ***
	14	***CHI3L1***	Chitinase 3 Like 1	Catalyzes the hydrolysis of chitin, plays a role in inflammatory processes, regulating allergen sensitization, inflammatory cell apoptosis, dendritic cell accumulation and M2 macrophage differentiation	**2.39 ***
	15	***GPRC5B***	G Protein-Coupled Receptor Class C Group 5 Member B	Member of the type 3 G protein-coupled receptor family; may modulate insulin secretion; may link retinoid and G-protein signaling pathways	**2.37 ***
WAT	1	***CST2***	Cystatin SA	Secreted thiol protease inhibitor found at high levels in saliva, tears and seminal plasma	**2.38 ***
	2	***ACSS1***	Acyl-CoA Synthetase Short Chain Family Member 1	Mitochondrial acetyl-CoA synthetase enzyme	**2.26 ****
	3	***GALNT16***	Polypeptide N-Acetylgalactosaminyltransferase 16	Catalyzes the O-linked oligosaccharide biosynthesis	**2.15 ***
	4	***DCLK1***	Doublecortin Like Kinase 1	Member of the protein kinase superfamily and the doublecortin family, involved in several cellular processes including neuronal migration, retrograde transport, neuronal apoptosis and neurogenesis	**2.09 ***
	5	***LCE1C***	Late Cornified Envelope 1C	Precursor of the cornified envelope of the stratum corneum	**1.96 ***
	6	***PCBP3***	Poly(RC) Binding Protein 3	Single-stranded nucleic acid binding protein, important for post-transcriptional modifications	**1.90 ***
	7	***LBX2***	Ladybird Homeobox 2	Putative transcription factor	**1.83 ****
	8	***KRTAP10-9***	Keratin Associated Protein 10-9	Essential for the formation of a rigid and resistant hair shaft, builds interfilamentous matrix for keratin filaments	**1.83 ****
	9	***SLC25A3***	Solute Carrier Family 25 Member 3	Transport of phosphate groups from the cytosol to the mitochondrial matrix	**1.82 ***
	10	***CLEC18A***	C-Type Lectin Domain Family 18 Member A	Binds polysaccharides in a Ca(2+)-independent manner, may be involved in cell adhesion, immune response and apoptosis	**1.82 ***
	11	***KIR2DS5***	Killer Cell Immunoglobulin Like Receptor, Two Ig Domains And Short Cytoplasmic Tail 5	Transmembrane glycoprotein, receptor on natural killer (NK) cells for HLA-C alleles, assumed to play an important role in regulation of the immune response	**1.81 ***
	12	***ZNF132***	Zinc Finger Protein 132	May be involved in transcriptional regulation	**1.80 ****
	13	***PAGE5***	PAGE Family Member 5	expressed in a variety of tumors and in some fetal and reproductive tissues, may protect cells from programmed cell death	**1.79 ***
	14	***ROR2***	Receptor Tyrosine Kinase Like Orphan Receptor 2	Tyrosine-protein kinase receptor which may be involved in the early formation of the chondrocytes	**1.75 ***
	15	***FDXR***	Ferredoxin Reductase	Mitochondrial flavoprotein; serves as the first electron transfer protein in all the mitochondrial P450 systems	**1.75 ***

Supplementary Table S1: Top 15 genes upregulated by heparin (≥ 1.5 fold) for each tissue ranked from highest to lowest. *(FC = fold change, * $p < 0.05$, ** $p < 0.01$, *** $p < 0.001$).*

	#	Gene name		Function according to Entrez Gene Summary	FC
**BM**	1	***ABI3BP***	ABI Family Member 3 Binding Protein	Protein-coding gene known to bind glycosaminoglycans	**-3.07 ****
	2	***GREM1***	Gremlin 1, DAN Family BMP Antagonist	BMP antagonist: inhibits BMP2 and downregulates BMP4-signaling; required for early limb development; inhibitor of monocyte chemotaxis	**-2.98 ***
	3	***CCKAR***	Cholecystokinin A Receptor	Member of the cholecystokinin receptor group of G-protein-coupled receptors; important for pancreatic exocrine secretion, gall bladder contractility and GI motility	**-2.91 ***
	4	***ACAN***	Aggrecan	Major component of extracellular matrix of cartilagenous tissues	**-2.35 ***
	5	***CALB2***	Calbindin 2	Intracellular calcium-binding protein belonging to the troponin C superfamily; abundant in auditory neurons	**-2.31 ****
	6	***MFAP5***	Microfibril Associated Protein 5	Microfibril-associated glycoprotein; component of microfibrils of the extracellular matrix; promotes attachment of cells to microfibrils	**-2.14 *****
	7	***IQGAP3***	IQ Motif Containing GTPase Activating Protein 3	Protein coding gene binding calmodulin and Ras GTPase	**-2.11 ***
	8	***DGKI***	Diacylglycerol Kinase Iota	Member of the type IV diacylglycerol kinase subfamily, which regulate the intracellular concentration of diacylglycerol	**-2.07 ****
	9	***DYSF***	Dysferlin	Belongs to the ferlin family and is a skeletal muscle protein; involved in muscle contraction; Plays a role in the sarcolemma repair mechanism of both skeletal muscle and cardiomyocytes	**-2.04 ***
	10	***GREM2***	Gremlin 2, DAN Family BMP Antagonist	Like GREM1 a cytokine that inhibits the activity of BMP2 and BMP4, thereby modulating the signaling by BMP family members	**-2.03 ****
	11	***FAM180A***	Family With Sequence Similarity 180 Member A	Protein coding gene	**-1.98 ***
	12	***HMGA1***	High Mobility Group AT-Hook 1	Encodes a chromatin-associated protein involved in the regulation of gene transcription, integration of retroviruses into chromosomes, and the metastatic progression of cancer cells	**-1.91 ***
	13	***PCOLCE2***	Procollagen C-Endopeptidase Enhancer 2	Binds to the C-terminal pro-peptide of types I and II procollagens, heparin-binding protein	**-1.90 ***
	14	***EPGN***	Epithelial Mitogen	Member of the epidermal growth factor family; promotes the growth of epithelial cells	**-1.89 ***
	15	***DLX3***	Distal-Less Homeobox 3	Member of Distal-less (Dlx) family of genes; Likely to play a regulatory role in the development of the ventral forebrain	**-1.86 ***
**UC**	1	***CALB2***	Calbindin 2	Intracellular calcium-binding protein belonging to the troponin C superfamily; abundant in auditory neurons	**-4.64 ***
	2	***P2RX1***	Purinergic Receptor P2X 1	Belongs to the P2X family of G-protein-coupled receptors, which function as ATP-gated ion channels and mediate rapid and selective permeability to cations	**-4.35 ****
	3	***SULT1E1***	Sulfotransferase Family 1E Member 1	Cytosolic enzyme; Sulfotransferase enzymes catalyze the sulfate conjugation of many hormones, neurotransmitters, drugs, and xenobiotic compounds	**-3.96 ***
	4	***IL11***	Interleukin 11	Member of the gp130 family of cytokines; stimulates the proliferation of hematopoietic stem cells and megakaryocyte progenitor cells; induces megakaryocyte maturation, which results in enhanced platelet production	**-3.91 ***
	5	***NEFM***	Neurofilament Medium	Type IV intermediate filament; encodes the medium neurofilament protein. Neurofilaments comprise the axoskeleton and functionally maintain neuronal caliber. They may also play a role in intracellular transport to axons and dendrites. This protein is commonly used as a biomarker of neuronal damage	**-3.70 ***
	6	***TNFSF15***	TNF Superfamily Member 15	Cytokine that belongs to the tumor necrosis factor (TNF) ligand family; expressed in endothelial cells; ligand for receptor TNFRSF25 and decoy receptor TNFRSF21/DR6; inhibits endothelial cell proliferation, and thus may function as an angiogenesis inhibitor	**-3.01 ***

	7	***PI16***	Peptidase Inhibitor 16	Protein coding gene, may inhibit cardiomyocyte growth	**-2.89 ***
	8	***ATP2A3***	ATPase Sarcoplasmic/Endoplasmic Reticulum Ca2+ Transporting 3	Encodes one of the SERCA Ca(2+)-ATPases, which are intracellular pumps located in the sarcoplasmic or endoplasmic reticula of muscle cells; catalyzes the hydrolysis of ATP	**-2.76 ***
	9	***NPTX1***	Neuronal Pentraxin 1	Member of the neuronal pentraxin gene family; human form is only localized in the nervous system; involved in mediating uptake of synaptic material during synapse remodeling	**-2.75 ****
	10	***GABBR2***	Gamma-Aminobutyric Acid Type B Receptor Subunit 2	belongs to the G-protein coupled receptor 3 family and GABA-B receptor subfamily; GABA-B receptors inhibit neuronal activity through G protein-coupled second-messenger systems	**-2.74 ***
	11	***EFR3B***	EFR3 Homolog B	Protein coding gene; component of a complex required to localize phosphatidylinositol 4-kinase (PI4K) to the plasma membrane	**-2.67 *****
	12	***SIK1***	Salt Inducible Kinase 1	Member of the adenosine monophosphate-activated kinase (AMPK) subfamily of kinases; Serine/threonine-protein kinase involved in several processes such as cell cycle regulation, gluconeogenesis, lipogenesis, muscle growth and differentiation and tumor suppression	**-2.67 ***
	13	***MYOCD***	Myocardin	Nuclear protein, which is expressed in heart, aorta, and in smooth muscle cell-containing tissues; functions as a transcriptional co-activator of serum response factor (SRF); plays a role in cardiogenesis and differentiation of the smooth muscle cell lineage	**-2.45 ****
	14	***INHBA***	Inhibin Beta A Subunit	Member of the TGF-beta (transforming growth factor-beta) superfamily of proteins; inhibits follicle stimulating hormone secretion from the pituitary gland	**-2.36 ***
	15	***XYLT1***	Xylosyltransferase 1	Xylosyltransferase enzyme; catalyzes the first step in biosynthesis of glycosaminoglycan: catalyzes transfer of UDP-xylose to serine residues of an acceptor protein substrate	**-2.32 ***
**WAT**	1	***CEP295NL***	CEP295 N-Terminal Like	Protein coding gene	**-2.26 ***
	2	***CYP2C19***	Cytochrome P450 Family 2 Subfamily C Member 19	Member of the cytochrome P450 superfamily of enzymes; localizes to the endoplasmic reticulum and is known to metabolize many xenobiotics such as barbiturates	**-2.08 ****
	3	***FBXL22***	F-Box And Leucine Rich Repeat Protein 22	Member of the F-box protein family; interacts with S-phase kinase-associated protein 1A and cullin in order to form SCF complexes which function as ubiquitin ligases	**-2.08 ***
	4	***ENC1***	Ectodermal-Neural Cortex 1	Member of the kelch-related family of actin-binding proteins; involved in the regulation of neuronal process formation and in differentiation of neural crest cells	**-1,92 ***
	5	***MORC3***	MORC Family CW-Type Zinc Finger 3	Localizes to the nuclear matrix and forms nuclear bodies via an ATP-dependent mechanism; predicted to have coiled-coil and zinc finger domains and has RNA binding activity	**-1,85 ***
	6	***MURC***	Caveolae Associated Protein 4	Promotes Rho/ROCK (Rho-kinase) signaling in cardiac muscles cells, and may facilitate myofibrillar organization	**-1,84 ***
	7	***MFAP3***	Microfibril Associated Protein 3	Component of the elastin-associated microfibrils	**-1,76 ***
	8	***NOTCH3***	Notch 3	Functions as a receptor for membrane-bound ligands Jagged1, Jagged2 and Delta1 to regulate cell-fate determination	**-1,73 ***
	9	***EPHA2***	EPH Receptor A2	Belongs to the ephrin receptor subfamily of the protein-tyrosine kinase family; have been implicated in mediating developmental events, particularly in the nervous system	**-1,73 ***
	10	***GOLGA8S***	Golgin A8 Family Member G	Protein coding gene; golgins constitute a family of proteins localized to the Golgi. This gene encodes a golgin, which structurally resembles its family member GOLGA2	**-1,72 ***
	11	***FKSG49***	Hepatocellular Carcinoma-Associated Antigen HCA25b	Protein coding gene	**-1.71 ***
	12	***SPDYA***	Speedy/RINGO Cell Cycle Regulator Family Member A	Regulates the G1/S phase transition of the cell cycle by binding and activating CDK1 and CDK2	**-1.69 ***
	13	***ASPHD1***	Aspartate Beta-Hydroxylase Domain Containing 1	Protein Coding gene with dioxygenase activity	**-1.67 ***

	14	***FFAR3***	Free Fatty Acid Receptor 3	G protein-coupled receptor that is activated by a major product of dietary fiber digestion; plays a role in the regulation of whole-body energy homeostasis and in intestinal immunity	**-1.66 ***
	15	***KRT16***	Keratin 16	Member of the keratin gene family; regulator of innate immunity in response to skin barrier breach - required for inflammatory checkpoints for the skin barrier maintenance	**-1.65 ***

Supplementary Table S2: Top 15 genes downregulated by heparin (≤ 1.5 fold) for each tissue ranked from highest to lowest.

*(FC = fold change, * p < 0.05, ** p < 0.01, *** p < 0.001).*

BM			UC			WAT		
Gene name	FC microarray	FC qRT-PCR	ID	FC microarray	FC qRT-PCR	Gene name	FC microarray	FC qRT-PCR
CD74	2.44 ***	6.97 *	*BMP4*	2.19 *	2.66 **	*FER1L6*	1.70 *	3.22 *
CFI	2.39 **	2.17 **	*WNT2*	5.20 **	4.83 *	*ANGPT1*	1.25 *	2.00 **
SVEP1	2.34 *	2.82 *	*COLEC12*	3.94 *	4.94 **	*RARRES3*	1.73 *	2.40 **
ITGB8	2.04 **	2.66 **	*VCAM1*	3.31 *	10.52 *	*HRAS*	1.66 *	2.12 **
BMP4	1.96 *	4.85 *	*ITGB8*	2.57 **	3.11 *	*KLF4*	1.60 *	2.05 ***
SEPP1	1.96 *	2.47 *	*DACH1*	2.56 **	3.62 *	*ACSS1*	2.26 **	1.64 *
ANKRD29	1.78 *	2.27 *	*CFI*	2.18 **	2.72 **	*HES5*	1.33 *	1.35 *
CFB	1.75 *	1.35 *	*SEPP1*	1.96 ***	2.98 **	*TUBB2B*	1.68 *	2.56 *
PDGFD	2.57 *	3.07 *	*ICAM1*	1.93 **	4.99 *	*MURC*	-1.84 *	-2.48 **
CALB2	-2.31 **	-4.26 **	*ARHGEF3*	1.76 *	3.76 *	*MORC3*	-1.85 *	-2.56 *
ABI3BP	-3.07 **	-3.61 *	*SVEP1*	1.67 **	2.40 ***	*NOTCH3*	-1.73 *	-2.20 *
GREM1	-2.98 *	-4.47 *	*GUCY1B3*	1.61 **	2.12 **	*FBXL22*	-2.08 *	-1.90 *
CCKAR	-2.91 *	-2.82 *	*CALB2*	-4.64 *	-7.62 ***			
NOTCH3	-1.60 **	-2.38 *	*IL11*	-3.91 *	-8.11 ***			
ID3	-1.55 *	-1.45 *	*SULTE1*	-3.96 **	-6.70 *			
			P2RX1	-4.35 **	-8.2 *			

Supplementary Table S3: Significant changes in gene expression of selected target genes induced by heparin. *Mean fold changes (FC) of microarray in comparison to FC determined by quantitative RT-PCR. (Upregulation by heparin in red, downregulation in blue.* $p < 0.05$, ** $p < 0.01$, *** $p < 0.001$).*

STC source	*Protein upregulation (≥ 20%)*	*Protein downregulation (≤ 20%)*
BM	43	13
UC	22	15
WAT	23	13

Supplementary Table S4: *Total number of proteins per source being altered at least ± 20% due to heparin treatment.*

STC source	*Protein upregulation (≥ 20%)*	*Protein downregulation (≤ 20%)*
TGFbeta pathway (25 members)		
BM	15	4
UC	10	4
WAT	9	6
WNT pathway (60 members)		
BM	22	7
UC	12	8
WAT	9	8
NOTCH pathway (20 members)		
BM	3	1
UC	2	1
WAT	2	2
PDGF pathway (49 members)		
BM	22	6
UC	10	3
WAT	13	6

Supplementary Table S5: *Heparin influences protein expression profiles of selected signaling pathways. The number of proteins of each tissue source and pathway with expression levels altered at least ± 20% due to heparin treatment are shown.*

Gene	Primer	Sequence (5'-3')
ABI3BP	forward	CCAGGTCTTAATGAAACTACTG
ABI Family Member 3 Binding Protein	reverse	AGCTAATTGTGTCTTGAGTG
ACSS1	forward	ATCAATGATGCCAAGTGC
Acyl-CoA Synthetase Short Chain Family Member 1	reverse	TCATCCACTATTTTCTTCAGC
ANGPT1	forward	GGTGTTTTGCAATATGGATG
Angiopoetin 1	reverse	TATTCCTTCCAGCCTCTTTG
ANKRD29	forward	TACAACTGCCCTATTCTTTG
Ankyrin Repeat Domain 29	reverse	CTTTGGTCCTAAATTCAGTGG
ARHGEF3	forward	CTCTCCATAATGACAGAACAAG
Rho Guanine Nucleotide Exchange Factor 3	reverse	ATCAGGCTTCCTAACATCTC
BMP4	forward	AGAACATCTGGAGAACATCC
Bone Morphogenic Protein 4	reverse	AATGTTTATACGGTGGAAGC
CALB2	forward	CTACGGATGTTTGACTTGAAC
Calbindin 2	reverse	CCCTGAAATTTAAGCAGGAAG
CCKAR	forward	CGTACCCCATTTATAGCAAC
Cholecystokinin A Receptor	reverse	ATCATTTGGCAGTAGAAAGC
CD74	forward	GAGTCACTGGAACTGGAG
CD74 Molecule	reverse	CATGGGATGAGGTACAGG
CFB	forward	AAGCCAAGATATGGTCTAGTG
Complement Factor B	reverse	TGTTAGTCCCTGACTTCAAC
CFI	forward	CAAGGTGCTGATACTCAAAG
Complement Factor I	reverse	TGGTAACCCATAGTTCTTCTC
COLEC12	forward	CTATGATGACAAGCTCACAG
Collectin Subfamily Member 12	reverse	TCTAGAATGTCTGATCTGAAGG
DACH1	forward	AGAGGCTAAAGAAGGAGAAG
Dachshund Family Transcription Factor 1	reverse	GTCATTTAAGACCCTGAGAC
FBXL22	forward	ATTGCTCAAATAGGGAAACG
F-Box And Leucine Rich Repeat Protein 22	reverse	TACCAGTTACCTGTTGTCTC
FER1L6	forward	GAGGTCAGATCCCAAAATTATC
Fer-1 Like Family Member 6	reverse	ATAAAATGGGCTGTTGGTTC
GREM1	forward	GATTGATCTAAGGGCCAAAG
Gremlin 1, DAN Family BMP Antagonist	reverse	CTAGGAGGCTGAGAAGATAC
GUCY1B3	forward	AAGGACTCATTTTGCACTAC
Guanylate Cyclase 1 Soluble Subunit Beta 1	reverse	CCTTCATGTCTATTTCAGTGC
HES5	forward	AAGAGAAAAACCGACTGC
Hes Family BHLH Transcription Factor 5	reverse	TTCTCCAGCTTGGAGTTG
HRAS	forward	ACCATTTTGTGGACGAATAC
HRas Proto-Oncogene, GTPase	reverse	AAGACTTGGTGTTGTTGATG
ICAM1	forward	CTCCAGACCTTTGTCCTG
Intercellular Adhesion Molecule 1	reverse	GTGGGGTTCAACCTCTG
ID3	forward	TCATCGACTACATTCTCGAC
Inhibitor Of DNA Binding 3, HLH Protein	reverse	CTTTTGTCGTTGGAGATGAC
IL11	forward	ACAGGGAAGGGTTAAAGG
Interleukin 11, Adipogenesis Inhibitory Factor	reverse	CAAACACAGTTCATGTCCC
ITGB8	forward	CAGTGACTACAATTTAGACTGC
Integrin Subunit Beta 8	reverse	TCCAGAGATCTTCTGTCTATG
KLF4	forward	TCTCCAATTCGCTGACCCATCCTCC
Kruppel Like Factor 4	reverse	AGGGAGCCGTCGGAGGGGGAGCG
MORC3	forward	TGTAGCTCGCTCAAGATGGC
MORC Family CW-Type Zinc Finger 3	reverse	CTGAATGGCCAGGTGTGACT
MURC	forward	CCTGTTGCCTGTTATCAAGCTGAC
Caveolae Associated Protein 4	reverse	GACACTGGAAACCTCTGATATGAC
NOTCH3	forward	CTACAATGGTGATAACTGTGAG
Notch3, Neurogenic Locus Notch Homolog Protein 3	reverse	CAGTCATCCTCATTAATCTCG
P2RX1	forward	CTTCAAGGTGTTTGGGATTC
Purinergic Receptor P2X 1	reverse	GTCATTGTAGGGATGATGTC

PDGFD Platelet Derived Growth Factor D	forward	TGACTTGTACCGAAGAGATG
	reverse	ACTAGCTGTATCCGTGTATTC
RARRES3 Retinoic Acid Receptor Responder 3	forward	CTGTAAACAGGTGGAAAAGG
	reverse	GTATCTCCTAATCGCAAAAGAG
SEPP1 Selenoprotein P	forward	TAAATATCAGAGTGTGCTGC
	reverse	TTAAACAACCACTTCCAACG
SPRY1 Sprouty RTK Signaling Antagonist 1	forward	CATAGACAAATCCTTGCTTAGG
	reverse	AAATCCTTTTCGGACAATCC
SULT1E1 Sulfotransferase Family 1E Member 1	forward	AGAGGATATCAGAAAAGAGGTG
	reverse	TGGTTCATAATTTCGTCTGG
SVEP1 Sushi, Von Willebrand Factor Type A, EGF and Pentraxin Domain Containing 1	forward	GTAAGAACATGTCCTCATCTC
	reverse	TTATCACTGCCTTCTAGTCTG
TUBB2B Tubulin Beta 2B Class IIb	forward	GGTAACAAATATGTTCCTCGG
	reverse	CGAAATTGTCTGGTCTGAAG
VCAM1 Vascular Cell Adhesion Molecule 1	forward	ACTTGATGTTCAAGGAAGAG
	reverse	TCCAGTTGAACATATCAAGC
WNT2 Wnt Family Member 2	forward	TTAATATGAACGCCCCTCTC
	reverse	TACCACCATGAAGAGTTGAC

169 ***Supplementary Table S6:*** *Quantitative RT-PCR primer sequences.*
